# Therapeutic Acellular Scaffolds for Limiting Left Ventricular Remodelling-Current Status and Future Directions

**DOI:** 10.3390/ijms222313054

**Published:** 2021-12-02

**Authors:** Sadia Perveen, Daniela Rossin, Emanuela Vitale, Rachele Rosso, Roberto Vanni, Caterina Cristallini, Raffaella Rastaldo, Claudia Giachino

**Affiliations:** 1 Department of Clinical and Biological Sciences, University of Turin, 10043 Orbassano, Italy; sadia.perveen@unito.it (S.P.); d.rossin@unito.it (D.R.); emanuela.vitale@unito.it (E.V.); rachele.rosso@unito.it (R.R.); roberto.vanni@edu.unito.it (R.V.); claudia.giachino@unito.it (C.G.); 2 Institute for Chemical and Physical Processes, CNR, 56126 Pisa, Italy; caterina.cristallini@cnr.it

**Keywords:** acellular scaffolds, myocardial infarction, ventricular remodelling, extracellular matrix, cardiac regeneration, tissue engineering, nanoparticles, conductive polymers

## Abstract

Myocardial infarction (MI) is one of the leading causes of heart-related deaths worldwide. Following MI, the hypoxic microenvironment triggers apoptosis, disrupts the extracellular matrix and forms a non-functional scar that leads towards adverse left ventricular (LV) remodelling. If left untreated this eventually leads to heart failure. Besides extensive advancement in medical therapy, complete functional recovery is never accomplished, as the heart possesses limited regenerative ability. In recent decades, the focus has shifted towards tissue engineering and regenerative strategies that provide an attractive option to improve cardiac regeneration, limit adverse LV remodelling and restore function in an infarcted heart. Acellular scaffolds possess attractive features that have made them a promising therapeutic candidate. Their application in infarcted areas has been shown to improve LV remodelling and enhance functional recovery in post-MI hearts. This review will summarise the updates on acellular scaffolds developed and tested in pre-clinical and clinical scenarios in the past five years with a focus on their ability to overcome damage caused by MI. It will also describe how acellular scaffolds alone or in combination with biomolecules have been employed for MI treatment. A better understanding of acellular scaffolds potentialities may guide the development of customised and optimised therapeutic strategies for MI treatment.

## 1. Introduction

Myocardial infarction (MI) is responsible for major cardiovascular associated death worldwide [1]. Following MI, inflammatory responses become activated to induce cardiomyocytes (CMs) death and degrade extracellular matrix (ECM) within a few days leading towards adverse left ventricular (LV) remodelling within a few weeks. If left untreated, it can ultimately result in heart failure (HF). Ventricular remodelling is a compensatory pathological outcome triggered by MI. It consists of shape, size and function alteration of the heart, mainly due to the wall thinning and dilation accompanied by an impairment in cardiac electrical conductance [2]. Commonly used approaches to mitigate ventricular remodelling are drug treatments, heart transplants and various surgical and non-surgical approaches. The main current medical therapies include angiotensin-converting enzyme inhibitors, angiotensin receptor blockers, aldosterone antagonists, and β-adrenergic receptor blockers, but these drugs are incapable to recover functional infarcted myocardium [3]. Heart transplant is considered when damage caused by MI has led towards end-stage HF. However, it offers certain post-operative complications that include organ rejection, graft failure, side infections in addition to difficulty in finding donors [4]. Other approaches include surgical procedure coronary artery bypass graft (CABG) and non-surgical approach percutaneous coronary intervention (PCI) to revascularise infarcted myocardium. These treatments can decrease morbidity and mortality rates but are incapable to avoid ventricular remodelling and progressive fibrosis that ultimately leads towards HF [5,6]. In recent times, tissue engineering has come forward and has designed numerous effective therapeutic options to counteract various physiological conditions. Numerous tissue engineering strategies are under investigation to restore and improve cardiac function and myocardial defects post-MI. One of these includes scaffolds that are potential candidates to recover post-MI damage in the heart. Temporary and local application of therapeutic scaffolds are one of the most promising therapeutic interventions under development to overcome cardiac damage and ventricular remodelling caused by MI.

In this regard, formerly tissue engineering approaches have paid attention to leverage stem, precursor or differentiated CM cells-based approaches to resolve and restore infarcted myocardium and ventricular remodelling post-MI [7,8]. It has been demonstrated that cell-based approaches contribute to cardiac regeneration because of their ability to secrete cardioprotective and pro-regenerative molecules in infarcted regions [9]. These approaches in clinical translation have presented some difficulties and limitations related to uncertain efficacy. The engraftment rate of the transplanted cells in the infarcted cardiac region is very low (< 2%). The most common reason for the loss of transplanted cells is their poor retention and survival in the hostile ischemic local microenvironment of the infarcted myocardium [7].

Scaffolds are therapeutic materials whose specific composition can bring forth biological cues along with mechanical support; they include bioscaffolds, patches, matrices and hydrogels. The basic framework of scaffolds is synthesised from polymers whose physical and chemical properties can be easily controlled. However, such scaffolds alone possess low bioactivity and therefore their major functional benefits are limited to structural and mechanical support only.

Cell-based approaches coupled with scaffolds have improved to some extent the efficacy of transplanted cells in terms of retention but some concerns still exist: limited nutrient and oxygen supply to the cells loaded on the scaffold due for instance to an insufficient vascularisation as well as the risk of host tissue immune response that can compromise cell viability [8,10]. Moreover, the exogenous implanted stem cells are restrained to integrate and adhere in infarcted myocardium and also interfere with the homing and recruitment of endogenous stem cells [7,8,10,11,12,13]. These issues with cell-based tissue engineering approaches still require further scientific efforts.

**Figure 1:**
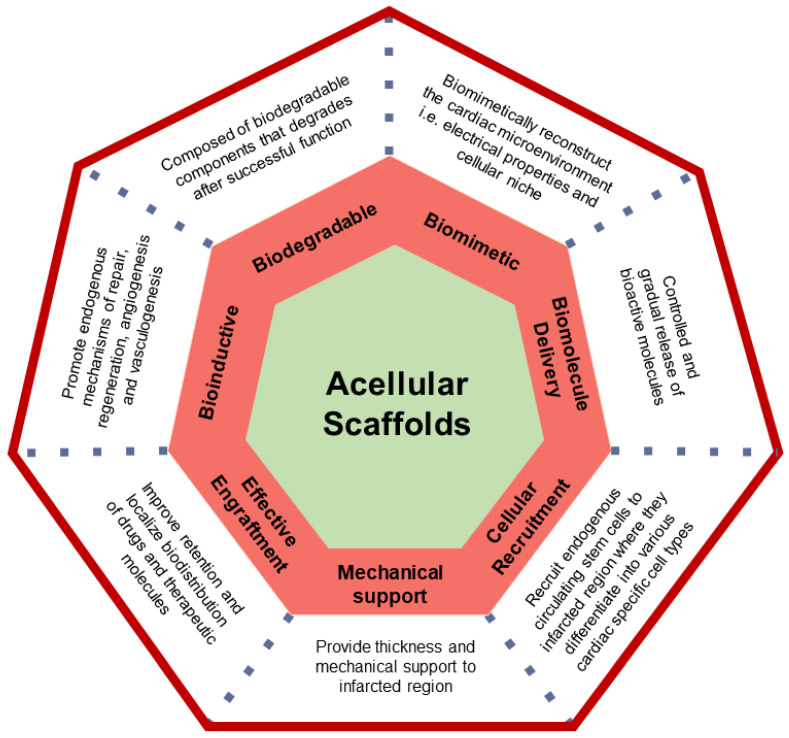
General characteristics of acellular scaffolds. Each characteristic is relevant to enable their use as therapeutic constructs to limit left ventricular remodelling.

On the other hand, functional acellular scaffolds coupled with biological molecules, drugs or other physiologically useful components are endowed with the potentiality to orient towards normal physiological function. They may encompass the key to unlock and elicit endogenous repair and regeneration processes at the infarcted site. Depending on their structural and functional targeted role, these acellular scaffolds can be architected to impart useful characteristics which include biodegradability, biocompatibility, biomimetism in addition to represent carriers and recruiters of useful endogenous and exogenous therapeutic molecules (Figure 1). To reduce ventricular remodelling after MI, the most important contributions of scaffolds are to provide temporary mechanical support as well as bioactive moieties and environmental cues to encourage physiological healing and regenerative processes [14]. In this regard, acellular scaffolds can be enriched with ECM components, cardioprotective agents, growth factors, peptides, recombinant proteins, enzyme inhibitors, nucleic acids, and drugs to impart unique beneficial properties.

This review summarises the studies conducted in the past five years on acellular scaffolds as therapeutic candidates to overcome post-MI ventricular remodelling. Indeed, a comprehensive understanding of this promising therapeutic approach can pave its path to clinical translation.

## 2. Non-Functionalised Acellular Scaffolds

Acellular scaffolds with biological, polymeric or bio-artificial nature, endowed or not with electrical cues, have been developed and tested both in vitro and in vivo (Table 1).

### 2.1. Biological Acellular Scaffolds

ECM provides structural support to cardiac tissue and is also involved in the integration of extracellular signals and the regulation of morphology, migration, differentiation and proliferation of cardiac cells [15,16]. After MI, dynamic changes in ECM drive endogenous inflammation, repair and regeneration [15,17]. The application of biological ECM on infarcted hearts can potentially improve endogenous repair and regeneration.

Acellular scaffolds with physiological anisotropy and integrated bioactive ECM can be potential candidates to provide a favourable and conducive microenvironment to recruit host cells and restore normal function [18]. The strategy, using decellularised tissue-derived ECM as acellular patches, presents a promising benefit due to its biological nature. The decellularisation step eliminates the cellular components and maintains some degree of ECM integrity in terms of its bioactivity, native three-dimensional (3D) architecture and surface structural composition [19]. After decellularisation, it has been suggested that numerous ECM factors, cell binding ligands and cytokines may be retained that can offer an environment suitable to allow cell integration, adhesion and interaction [20].

The therapeutic potential of decellularised porcine myocardium slices (dPMS) as acellular scaffolds was investigated in a rat MI model by Shah & Zhang group. dPMS scaffolds with different thicknesses (300 and 600 μm) were applied to the infarcted myocardium. In both groups, dPMS scaffolds were tightly affixed to the myocardium that provided strong LV mechanical support and impeded thinning of the LV wall. Histological analysis demonstrated that dPMS was endured as a whole patch unified with host myocardium with no gap. Moreover, it also contributed effectively to host cell infiltration, formation of vessels and improvement in cardiac function. At 4 weeks, in comparison to 300 μm thickness dPMS, 600 μm patch was more effective in preventing thinning of LV wall and fractional shortening (FS). In addition to this, the 300 µm thickness patch degraded significantly as observed after 4 weeks of transplantation thus highlighting that the durability of dPMS can be compromised in long-term applications [21]. Unlike other patches, the capability of dPMS to attach to host tissues might represent a unique advantage. Other patches require additional adhesive materials like fibrin glue in between patch and host tissue to aid the binding of the patch into the host myocardium [22]. dPMS itself displays adhering ability at the implantation site and, moreover, after transplantation, it also maintains its physical integrity. In decellularised tissues, numerous growth factors are retained that additionally furnish bioactivity to acellular ECM [19]. However, the amount of growth factors trapped in decellularised tissues is considerably lower in comparison with the native cardiac tissue [23]. One of the strategies to tune dPMS degradation is the use of natural crosslinkers, such as genipin, a water-soluble low-toxic crosslinker extracted from gardenia fruits able to prolong the matrix hydrogel degradation rate [24]. dPMS retains numerous useful mechanical aspects that include considerably high tensile modulus and stiffness in both longitudinal and circumferential directions and the preservation of the anisotropic nonlinear nature of heart tissues [25,26]. Further, in vivo studies with tuned dPMS to improve its durability and imitation of native myocardium can more prominently highlight its beneficial aspects in treating MI. Another study prepared decellularised myocardial matrix hydrogel from porcine LV myocardial tissue and investigated its efficacy to counteract negative LV remodelling in a rat chronic MI model. The authors demonstrated that this hydrogel thickened LV apical wall, reduced fibrosis, upregulated cardiac muscle contraction-related gene expression and downregulated fibrotic pathway-related gene expression [27].

Porcine small intestine submucosa ECM (SIS-ECM) is a decellularised ECM structure derived from small intestinal submucosa that encompasses several useful and clinically operative characteristics. SIS-ECM has retained its native 3D structure and cell signalling cues that provide a favourable and encouraging homeostatic atmosphere to allow functional survival and retention of host cells. Moreover, it is absorbable and has preserved sufficient biological activity to regulate the endogenous host cells infiltration, differentiation, growth and proliferation. It shows another appealing feature that consists of the inability to provoke host immunological responses [28,29]. CorMatrix ECM (CorMatrix Cardiovascular, Inc., Roswell, GA, USA) is a commercially available SIS-ECM that has been employed for several cardiovascular uses [30]. A study detected the therapeutic functional impact of CorMatrix ECM in MI large animal models. Following 75 min of coronary ischemia, the CorMatrix ECM scaffold was applied to infarcted epicardium in swine. The cardiac function was monitored serially after one and six weeks by magnetic resonance imaging (MRI) and histological examination. Ventricular wall thickening increase, cardiac fibrosis prevention and vasculogenesis mainly of small arteries network in infarcted regions were the most prominent biological effects observed. However, the study was limited by small sample size [31].

The relevance of using an intact bioactive scaffold for post-MI functional recovery was highlighted by the comparison between CorMatrix-ECM and its biologically inactivated form. The latter was obtained by treating the bioactive scaffold with either glutaraldehyde cross-linking or by guanidine hydrochloride. Both bioactive and inactivated CorMatrix-ECM scaffolds were implanted in a rat MI model. The results confirmed that bioactivity within ECM acellular scaffold is crucial for its functional impact. In fact, after 6 weeks, it was shown that only bioactive ECM could promote vasculogenesis and repair. Moreover, in this study through in vitro assays, it was demonstrated that human cardiac fibroblasts and fibroblast growth factor-2 (FGF-2) efficiently interact with ECM scaffolds thus suggesting that the in vivo functional impact of ECM on vasculogenesis and stimulation of pro-repair processes, including enhanced activation of epicardial progenitor cells, could be mainly attributed to these endogenous components. Despite these interesting results, however, the authors pointed out that the chemical procedure of CorMatrix-ECM decellularisation caused cell toxicity [32]. The clinical feasibility and safety of CorMatrix-ECM was also assessed in a pilot, non-random and unblind open-label Phase 1 clinical trial (NCT02887768). In this study, CorMatrix-ECM was surgically implanted at the time of CABG surgery in MI patients. Within four weeks from acute myocardial ischemic injury, scaffolds were implanted in patients and the clinical effects were studied serially up to six months through CMR imaging. It was observed that CorMatrix-ECM is feasible and safe to use and also improved tissue perfusion in human patients. However, the clinical study was not powered enough to confirm its functional effectiveness as it was limited to eight patients with MI and among those only two subjects had evident cardiac injuries before scaffold implantation [33].

VentriGel is an ECM injectable hydrogel that is derived from decellularised porcine myocardium. Unlike other biological scaffolds that acted as mechanical support, the VentriGel was designed for in situ assembling into a fibrous and porous scaffold that provides an ECM microenvironment, to recruit endogenous cells. In terms of stiffness, it is weak as it has two-fold lower stiffness than healthy myocardium, yet this feature allows its less invasive delivery through a catheter [34,35]. A safety and feasibility trial of VentriGel (NCT02305602) has been conducted in a clinical setup. VentriGel was injected in the infarct zone in both early and late post-MI patients with LV dysfunction already treated with PCI and follow-up was performed for six months. VentriGel was safe and well-tolerated. Indeed, this new approach paved the way to further investigation with injectable biological scaffolds in a clinical setup. The main goal of this study was limited to the assessment of clinical feasibility, however other effects in terms of efficacy were also noticed that included an improvement in the quality of life as indicated by the increase in Six-Minute Walk Test distance and improved scores of New York Heart Association functional class. The authors, however, observed scarce effects on the limitation of adverse cardiac remodelling. In fact, MRI analysis showed a decrease in both LV end-systolic volumes (LVESV) and end-diastolic volumes (LVEDV) with consequent unchanged ejection fraction (EF). Moreover, VentriGel did not affect the infarct size [36]. Since the authors stated that results were not fully significant likely due to the limited number of recruited patients, VentriGel should be further investigated in larger random and controlled clinical studies. However, injectable ECM materials could have some limitations due to their low spreading which can induce a disruption of gap junctional cellular communication among the CMs in the site of injection, thus potentially causing an alteration of action potential propagation with consequently enhanced arrhythmia vulnerability [37].

Several cardiac ECM therapies exploit adult animal cardiac tissues to derive ECM. Adult mammalian cardiac tissues, however, possess limited regenerative potential. In contrast, the cardiac tissues of neonates can undergo favourable and robust cardiac regeneration. It is therefore hypothesised that ECM derived from neonatal tissues might be more effective therapeutically in comparison to adult tissue-derived ECM. A study in an adult mice acute MI model compared the therapeutic potential of ECM derived from neonatal mouse heart (nmECM) versus ECM derived from adult mouse heart and concluded that nmECM can more effectively improve cardiac function and averts widespread adverse LV remodelling [38].

In recent years, placenta and amniotic membrane (AM) are used in surgical regenerative medicine, originally because they are a good source of juvenile cells [39]. It has been determined that amniotic epithelial cells induce epithelial-to-mesenchymal transition (EMT) in the ventricular remodelling process [40]. In a study on rodent MI models, decellularised human amniotic membrane has been demonstrated to improve cardiac function following MI by improving cardiac contractility and decreasing fibrosis and scar size [41]. Another study determined the impact of naïve and processed AM on post-MI LV remodelling. The study employed unmodified human AM after EMT (EMT-AM) and decellularised AM (Decell-AM) and evaluated their impact on the mice MI model. After four weeks of implantation, it was demonstrated that in this xenogeneic model, Decell-AM is more beneficial; indeed, it decreased infarct size, increased scar thickness and improved LV contractile function. Unmodified AM, with or without EMT induction, provoked local immune responses that averted relevant recovery in cardiac function and was also not firmly attached to the epicardium. Decell-AM was immunologically inert and affixed firmly to the infarcted epicardium that helped evade pathological post-MI ventricular remodelling and improved systolic and diastolic function. These data suggested that Decell-AM could be valuable to develop therapeutic epicardial strategies to counter post-MI dysfunction. This xenogeneic approach requires further elaborative research in pre-clinical models to completely understand its relevance as a clinically translatable candidate [42].

### 2.2. Polymeric Scaffolds

During the fabrication of scaffolds, specific architectural, biochemical and biophysical characteristics can be acquired through customisation of various factors such as mechanical properties, microstructure and cross-linking degree. For bioengineering applications, degradable polymers are employed frequently [43]. In recent years, numerous studies have architected scaffolds derived from either synthetic or natural polymers and ascertained their therapeutic efficacy to mitigate LV remodelling post-MI.

Poly-(ε-caprolactone) (PCL) is a synthetic FDA approved semi-crystalline aliphatic polyester with high thermal stability, biocompatibility, long-term biodegradability and elastomeric properties. Poly-(glycerol sebacate) (PGS) is a synthetic thermoset polymer well-known for its elastic, biodegradable and biocompatible characteristics that make it a useful candidate for soft tissues engineering and cardiac scaffold construction [44]. When elastomers are implanted in an in vivo mechanically dynamic environment, they present useful features that mimic mechanical characteristics of soft tissues thus allowing them to sustain and recover numerous deformations [45]. However, the processability of PGS alone in fibrous structure is limited, mainly by electrospinning technique, due to its low viscosity and chain entanglements, for this reason, PGS is blended with other polymers such as PCL [46]. A study constructed a hybrid scaffold composed of PGS and PCL polymer, possessing worthier mechanical characteristics owing to its well-organised stacked construction with regular crisscrossed filaments and interconnected micropores. Four weeks after scaffold implantation, the results demonstrated improvement in numerous aspects of myocardial remodelling that included decreased ventricular wall thinning, reduced infarct size, enhanced vascular density and increased M2 macrophage infiltration which may further contribute to the mitigation of myocardial apoptosis. In addition to this, another promising characteristic was that this PGS-PCL scaffold can be customised to the desired shape and can be easily compressed, folded and rolled that depicts its attractive applications owing to its use as an annular-shaped restraint device and coming up to the requirements for minimally invasive operations [46]. Biocompatible therapeutic hydrogels employed as scaffolds have shown various promising therapeutic responses to improve cardiac function and overcome LV remodelling post-MI. Pre-clinical studies have shown that hydrogels are not suitable for delivery through a catheter due to their fast gelation, high viscosity, embolic nature and hemocompatibility concerns [47,48]. In recent years, to circumvent this limitation freely floating progelator constituents are devised as cyclic peptides that tend to form rapid assembly into hydrogels through linearisation by disease-linked enzymes. Thus, they can be employed by means of low resistance injections in disease-specific regions [49]. Their successful in vivo use is assessed by their capability to be applied through cardiac injection catheter without presenting cytotoxicity, hemocompatibility and clogging concerns [50]. These studies have set the platform for the formulation of dynamic biomaterials in terms of both function and structure for post-MI cardiac damage.

Self-assembling peptides (SAPs) are synthetic multifaceted polymers that can be customised to develop bioactive scaffolds via biocompatible supramolecular interactions. Their composition includes a series of hydrophobic and hydrophilic amino acids that can be exploited for tailored structure organisation by adding multivalent ions. They can incorporate bioactive peptide motifs to the main oligopeptide core motif that forms hydrogel [51,52]. These hydrogels possess numerous attributes that include mild gelation, injectability, biocompatibility, biodegradability, prompt migration at the cellular level due to their flexible nature, small size that make them suitable for endothelial cell adhesion and angiogenesis, the resemblance with ECM and amenability to sequence alterations [52]. Recently a freely floating cyclic peptide progelators has been engineered that remains in solution unless activated by proteolytic means to generate SAPs that auto-assemble themselves into an elastic and viscous hydrogel [50]. This study has successfully demonstrated the ability of KFDF_Cyclic_ SAPs to promote activated hydrogel scaffold gelation in in vivo rat MI model. This simple approach has used a complex natural local MI microenvironment to deliver and assemble scaffolds, thus paving the pathway for other SAP molecules with such progelator approach for in vivo cardiac injection. Moreover, this strategy can be exploited to use in conjunction with numerous small therapeutic molecules such as drugs and short peptides. This can even have the potential for targeted delivery of large therapeutic molecules, such as growth factors, and cells by injection of their mixture with the progelators [50].

Bioactive motif seryl-aspartyl-lysyl-proline (SDKP) has been proven to hold pro-angiogenic, anti-inflammatory, and anti-fibrotic properties in various studies [53,54,55]. Firoozi and co-workers tailored SAP hydrogel by conjugating SDKP to core motif composed of four RADA sequences and evaluated therapeutic efficiency of this cell-free (RADA)4-SDKP hydrogel in rat MI model. SAP hydrogel (0.25%), alone or combined with bone marrow mesenchymal stem cells (BM-MSCs), was intramyocardially injected into the peri-infarct region. The authors found that this acellular SAP hydrogel was highly cardioprotective and safe to use. The therapeutic outcomes included significantly reduced fibrosis, reduced infarct size, enhanced microvasculature, reduced inflammatory response and improvement in cardiac functional parameters as indicated by echocardiographic measurements. In addition to this, the authors also compared cell-free (RADA)4-SDKP hydrogel with BM-MSCs loaded (RADA)4-SDKP hydrogel and interestingly found that the addition of BM-MSCs did not improve cardiac therapeutic outcomes of the (RADA)4-SDKP hydrogel alone. Taken together these results highlighted that cell-free (RADA)4-SDKP hydrogel significantly restore cardiac function and decelerate LV remodelling after MI in rats and could hold promising therapeutic potential as an acellular scaffold for MI treatment in clinical settings [56]. These results are encouraging, and most synthetic SAP injections are minimally invasive procedures, by exploiting the pathological phenomenon of leaky vasculature from the bloodstream into heart tissue [48]. However, SAP-based scaffolds still need further scientific evaluation to explore their functional impact in more elaborative pre-clinical studies, both in small and large animals, to completely understand their possible functional outcomes.

Besides scaffolds constructed from synthetic polymers, several natural scaffolds have been developed. Alginate is an example of a natural anionic polymer commonly obtained from brown seaweed. In the biomedical field, it has been investigated extensively owing to its biocompatibility, low toxicity and low cost. Moreover, when divalent cations such as Ca^2+^ are added to a solution of sodium alginate, mild gelation and crosslinking occur resulting in alginate hydrogels preparations. The disruption of ECM with high extracellular Ca^2+^ levels together with apoptosis are the major events in large MI. The combination of alginate solution with calcium gluconate also known as a bioabsorbable cardiac matrix (BCM) is a novel strategy to evade pathological remodelling as it provides transient structural support to infarcted areas till the development of mature fibrotic tissue [57]. Ca^2+^ presence in the infarcted zone enables cross-linking of BCM into elastic ECM like scaffolding material that forms support for cardiac tissue, and it degrades gradually with time and ultimately is eliminated through renal excretion. Alginate hydrogel structures mimic ECM of living tissues that make it very suitable for numerous biomedical uses that involve regenerative tissue engineering, bioactive molecules delivery, cell implantation and wound healing. Alginate hydrogels are minimally invasive and also enable the controlled release of biomolecules such as drugs and proteins [58]. These properties enable the use of alginate-based biomaterials as a medical therapy. In pre-clinical studies, it has been evaluated that alginate-based polymers provides a flexible scaffold for infarcted myocardium, replace degraded ECM, give a structural cast to damaged tissues, evade dyskinesis, increase LV wall thickness, alter LV geometry and improve indexes of LV systolic function [59,60]. A study tailored gelatine alginate hydrogel with unique capillary-like channels (Capgel) and injected it in an anteroseptal wall at the infarct border zone of the acute MI rat model to evaluate its therapeutic efficacy. It was found that Capgel is safe to use and in terms of therapeutic efficacy, it was shown to improve LV function and blood vessel formation at infarcted zone [61].

Relevance of alginate hydrogels has also been demonstrated in clinical scenarios where it has proven to be a candidate for significant improvement of cardiac function in patients with cardiopathies. However, in these first studies, a short follow up time was applied [62,63]. More recently, a clinical study named AUGMENT-HF (NCT01311791) investigated the use of Algisyl (injectable calcium alginate hydrogel) in patients with advanced HF and compared it with standard medical therapy (SMT) followed by a follow up of one year. This study comprised an international, open-labelled and randomised clinical trial with the enrolment of 78 patients with advanced HF already receiving SMT. Some patients were randomly assigned to obtain Algisyl injections in the mid LV wall during the surgical procedure. Among these, 58 patients that completed one year follow up as planned were with ischaemic (57.7%) or non-ischaemic (42.3%) HF. It was observed that Algisyl along with SMT is more beneficial than SMT alone to provide continuous one-year beneficial outcomes in terms of clinical status, symptoms and exercise capacity. However, in the Algisyl group, cardiac function parameters were not statistically significant when compared with patients in the control group. The major limitations included the inability to blind the trial and the small sample size. This study paved the way to evaluate the relevance of Algisyl in elaborative clinical assessments [64]. Another clinical study (NCT01226563) named PRESERVATION-I involved an international randomised, double-blind, controlled clinical trial encompassing 303 patients who have already undergone successful PCI of ST-segment elevation MI but still have large infarcted regions. BCM was injected into the infarct artery two to five days post-primary PCI and then follow-up was performed till six months through 3D echocardiography and quality-of-life assessments. However, the results highlighted no reduction in adverse LV remodelling or clinical benefits in large MI bearing subjects. There was an only improvement in the Six-Minute Walk Test. These disappointing results could be due to several reasons that may include BCM volume (4 mL) injected, possibly not adequate for large MI setting where it is very difficult to revert remodelling, BCM incapability to reach intracellular space in infarcted area, or linked microvascular occlusion that might have averted it to reach its target. Furthermore, it cannot be excluded that BCM had been injected in the infarct artery and not directly on the myocardium. The authors argued that BCM may suit MI patients having small infarcts with the risk of adverse LV remodelling; they also suggested that it is likely that early administration might provide some clinical benefits [65].

Hyaluronic acid (HA) is another natural polymer that is anionic, non-sulfated glycosaminoglycan, highly hydrophobic and immunologically inert. It has significant structural and functional roles in ECM architecture, cell signalling and angiogenesis. Its use as a biomaterial has gained wide acceptance in tissue engineering due to its biocompatible, biodegradable and chemical modifiability features [66]. Its use as a building block to synthesise cardiac therapeutic scaffolds to treat MI has been studied and accepted in the past [67,68]. The potential of HA hydrogel scaffold has been investigated in the ovine MI model where the scaffold exploited the tandem cross-linking strategy, where the first crosslinking between scaffold and host allowed the successful injection and retention whereas the second crosslinking in situ after injection provided stiffness to the hydrogel. The outcomes highlighted a significant improvement in cardiac function and ventricular geometry [69]. This study provided evidence of in vivo feasibility of modified HA hydrogels in a pre-clinical setup. However, the limited follow-up time could not clearly ascertain its long-term feasibility and efficacy.

A study reported a viscoelastic adhesive patch termed as gel-point adhesive patch (GPAP) composed of ionically cross-linked and transparent starch hydrogel [70]. This patch was designed by finite-element simulations of LV remodelling which enables relatively low dynamic modulus at gel point to equilibrate the fluid and solid properties of its material that resulted in its appealing therapeutic efficiency. The simulation guided designing approach imparted self-adaptive properties in a patch that enabled it to accommodate the cyclic deformation in the epicardium, arrhythmic heartbeat and sustain reliable efficiency to restrain ventricular dilatation and prevent adverse LV remodelling and HF after MI. This patch surpasses other cell-free patches in terms of its superior performance, in fact, it reversed LV remodelling and restored cardiac function after both acute and subacute MI in rats. The patch was also cost-effective, safe and immunologically inert. This study has set the path to fully investigate the translational benefit of GPAPs in large animal models and clinical studies. The simulation-based strategy established in this work might guide one to design personalised GPAPs in clinical scenarios based on subject data, infarcted zone, heart geometry, age and gender that might enable precise therapy along with enhanced simulation algorithms and fabrication methods of GPAP for customised requirements. Moreover, the mechanical design that balanced both fluidity and solidity could have improved the potential for mechanical support on cyclically loaded patches that commonly do not have a distinct deformation reference point [70].

Silk fibroin (SF) is an FDA-approved natural biomedical material that can be easily modified and possess very good mechanical stability and biocompatibility [71]. A study evaluated the therapeutic efficacy of SF hydrogel scaffolds with differences in biodegradation behaviour through peptide modification. Peptide modified SF (SF + Pep) hydrogel possesses a rapid degradation rate whereas unmodified SF hydrogel degrades slowly. The impact of hydrogel biodegradation on attenuating LV remodelling was assessed in the rat MI model for up to 12 weeks. It was observed that unmodified SF hydrogel prevented negative LV remodelling, including dense random alignment of collagen fibres, attenuation in LV enlargement, increase in LV wall thickness and improvement in echocardiographic parameters [72]. This study confirms the importance of a long-term permanence of biomaterial in the heart to obtain better outcomes.

Another FDA-approved natural biomaterial is chitosan (CS) hydrogel which has been widely used in tissue engineering, drug delivery and bone, cartilage, skin and nerve repair [73]. The degree of acetylation (DA) of CS influences its degradation and biological activity [74,75]. CS hydrogels hold optimum physicochemical, mechanical, and biodegradation properties to regenerate infarcted myocardium [76]. In preclinical acute MI rat models, 75% to 90% deacetylated 2% CS hydrogels have been shown to improve cardiac function and avert negative LV remodelling by reducing infarct size, promoting angiogenesis and improving LV function [77]. A study ascertained therapeutic impact of 3% w/w CS hydrogel patches with DA ranges 2.5% to 38% in murine MI model and found that CS hydrogels with 24% DA work best to prevent LV remodelling by improving LV function and decreasing fibrosis [78].

Other studies have reported hydrogel scaffolds prepared from various biocompatible polymeric materials that included N-isopropylacrylamide (NIPAAm), poly N-isopropylacrylamide (PNIPAAm), 2-hydroxyethyl methacrylate (HEMA), methacrylate polylactide (MAPLA), dextran (Dex), poly(ε-caprolactone) (PCL) and gelatine [79,80,81]. Poly (NIPAAm-co-HEMA-co-MAPLA) hydrogels [79] in an infarcted swine model, whereas Dex-PCL-HEMA/PNIPAAm (DPHP) hydrogels [80] and gelatine methacryloyl hydrogels [81] in infarcted rodents have also been shown to attenuate LV remodelling and improve cardiac function.

### 2.3. Conductive Scaffolds

Irregular electric conduction is a common after-effect of MI. The physiological changes after MI include the replacement of necrotic CMs by collagen tissues and fibroblasts. This excess in ECM deposition by fibroblasts causes non-uniform electrical conductivity and CM uncoupling, thus inducing CMs vulnerable to re-entry. This mechanism favours ventricular arrhythmias to occur, thus leading to an increased risk of sudden cardiac death [82,83]. In a healthy heart, myocardial conduction depends on the intercellular transfer of ions through gap junctions mainly located at the intercalated discs of CMs, thus leading to an anisotropic pattern of cardiac conductivity [84]. These cells express multiple transmembrane proteins called connexins (Cx) among which Cx-43 is the primary component that forms ventricular gap junction channels. The reduction of expression of Cx-43 and its lateralisation on CMs membranes takes place together with its inhibition, all these changes impair electric propagation and alter the anisotropic pattern of cardiac conductivity, thus promoting cardiac arrhythmias [84,85]. For this reason, it is essential for biomimetic and 471 operative cardiac recovery to restore to restore the normal electrical conduction in the infarcted heart. A conductive scaffold can connect isolated CMs to revive ventricular function and synchronise propagation and the subsequent contraction along with providing mechanical support [86].

A very recent report has shown that cardiac scaffold based on electrospun silk fibroin (rGO/silk) coupled with reduced graphene oxide (rGO) can be optimised to present improved saturability and anisotropic conductivity in normalising post-MI cardiac functions. Four weeks after MI in the rat model, scaffold capability to improve CMs survival, lessen arrhythmias, thicken LV wall and enhance pumping function in addition to prominent effects on angiogenesis in infarcted myocardium was highlighted. Moreover, this study also evidenced that isotropic conductive rGO/silk patches and nonconductive silk ones did not show promising results in recovering the electrical environment, thus emphasising that optimised anisotropic scaffold composition is essential to achieve desired therapeutic function [87]. Another recent study documented a biocompatible self-doped polymeric scaffold in physiological pH that renders it electrically active in tissues. This scaffold was composed of conductive poly-3-amino-4-methoxybenzoic acid (PAMB) coupled with non-conductive gelatine (G) cross-linked by carbodiimide. The conductive potential of PAMB-G hydrogel was 12 times greater than gelatine hydrogel. Microelectrode array studies further revealed that PAMB-G hydrogel imparted greater field potential amplitude on the heart and could also transmit current to electrically excite one heart placed at a distance from another heart as compared to gelatine hydrogel. Moreover, in vivo studies in rat models demonstrated that PAMB-G hydrogel injection could preserve cardiac function by reducing arrhythmias and synchronising cardiac contractions [88].

Coadministration of various strategies is appreciated as it produces promising synergistic therapeutic results. A study has combined the application of a self-adhesive conductive hydrogel patch and injectable hydrogel in an infarcted region. Hydrogel patch constituted a homogenous network of dopamine-gelatine (GelDA) conjugates and dopamine-functionalised polypyrrole (DA-Py). The injectable hydrogel formulated in situ involved the Schiff base reaction between oxidised sodium hyaluronic acid (HA-CHO) and hydrazided hyaluronic acid (HHA). The co-administration of two systems in the infarcted region, HA-CHO/HHA hydrogel intramyocardially with a subsequent painting of conductive GelDA/DA-Py hydrogel patch on the cardiac surface, could outturn promising improvement of signal propagation in addition to enhancement of angiogenesis and cardiac function [89].

Conductive poly-pyrrole-chitosan hydrogel (PPY-CHI)-based scaffold in preclinical MI animal model could produce considerably improved electrical conduction and synchronised cardiac contraction. In particular, narrowed QRS and QT intervals along with reduced fibrotic scar resistivity demonstrated more efficient conduction in post-MI hearts leading to a lower incidence of arrhythmias. The authors suggested that conductive polymers caused a reduction in arrhythmic events by acting on conduction velocity and/or tissue impedance in the scar tissue [90]. Similar effects were displayed by another conductive scaffold synthesised by graphene oxide gold nanosheets (GO-Au) into CS [91]. Besides, the controlled degradation nature, this combination showed an improved electrical conductivity to two folds along with a porous framework. On the other hand, when graphene oxide NPs were incorporated in oligo (PEG fumarate) (OPF) hydrogels, the resulting GO-OPF hydrogels displayed semiconductive nature only. However, 4 weeks after MI, in the group with GO-loaded hydrogel GAP junction remodelling was observed in the infarcted area and this result is consistent with the increase in Cx-43 expression attributed to the activation of the Wnt signal pathway. Moreover, CMs isolated from the infarcted region displayed an improvement in Ca^2+^ signal conduction and beating frequency. These effects also account for the synchronisation of CMs thus improving LV function [92]. Pyrrole and dopamine (both undergo Fe^3+^ triggered polymerisation in situ) hydrogels when painted in an infarcted rat heart surface-bound strongly and boosted electrophysiological signal conduction thus reviving cardiac function [93]. Improvement in cardiac conduction by exploiting conductive scaffolds is well studied in pre-clinical research; however, the conduction stability and consistency of these scaffolds in a dynamic mechanical cardiac environment are still needed to better establish this approach in pre-clinical research before introducing it in clinical scenarios. A well-customised approach might further highlight the functional outcomes of conductive scaffolds in MI treatment.

**Table 1:** Summary of non-functionalised acellular scaffolds to treat post-MI damage in pre-clinical and clinical models.

Scaffold Composition	Model	Protocol Followed to Induce MI	Post MI Timing of Scaffold Application	Follow Up	Biological Effects	Ref.
Biological Scaffolds						
Decellularised porcine myocardium	Male SD rats	Permanent LAD ligation	Immediately (implanted)	Up to 4 weeks	↑ Cell attachment viability and infiltration, ↑ Vasculogenesis, ↑ EF, ↑ FS	[21]
Myocardial matrix	Female SD rats	Ischemia reperfusion	8 weeks (injected)	Up to 5.5 weeks	= LVEDV, = LVESV, ↑ apical wall thickening, ↓ Fibrosis, ↑ Cardiac muscle contraction-related gene expression, ↓ Fibrotic pathway-related gene expression	[27]
CorMatrix ECM	Male Landrace pigs	Ischemia reperfusion	75 min (implanted)	Up to 6 weeks	↑ LV wall thickness, ↓ Scar formation, ↓ Fibrosis, ↑ Vasculogenesis	[31]
CorMatrix ECM	Male Fischer rats	Ischemia reperfusion	3 weeks (implanted)	Up to 14 weeks	↑ Vasculogenic paracrine response, Stimulation pro-reparative pathways, ↑ Blood vessel assembly	[32]
CorMatrix ECM	Clinical study (NCT02887768)		(implanted)	Up to 6 months	↓ Scar burden, ↓ Perfusion of infarcted myocardium	[33]
VentriGel	Clinical study (NCT02305602)		(injected)	Up to 6 months	↑ Exercise capacity, = EF, = infarct size	[36]
nmECM	Male BALB/cJ mice	Permanent LAD ligation	Immediately (injected)	Up to 6 weeks	↓ EDA, ↓ ESA, ↑ FAC, ↑ FS, ↑ EF, ↓ Fibrosis, ↑ Angiogenesis	[38]
Human AM	Female SD rats	Ischemia reperfusion	2 days (injected)	4 weeks	↑ EF, ↑ FS, ↓ Infarct size, ↓ Fibrosis	[41]
Decell-AM	Male BALB/c mice	Permanent LAD ligation	Immediately (implanted)	4 weeks	↓ Infarct size, ↑ Wall thickness, ↑ Contractile function	[42]
**Polymeric Scaffolds**						
PGS-PCL	Male SD rats	Permanent LAD ligation	2 days (implanted)	Up to 4 weeks	↓ Ventricular wall thinning, ↓ Infarct size, ↓ Apoptosis, ↑ Vascular density, ↑ M2 macrophage infiltration	[46]
SAP-(RADA)4-SDKP	Adult male SD rats	Permanent LAD ligation	Immediately (injected)	4 weeks	↓ Fibrosis, ↑ Microvasculature, ↓ Inflammatory response, ↓ Infarct size, ↑ EF, ↑ FS, = LVEDD, ↓ LVESD	[56]
Capgel	Male SD rats	Permanent LAD ligation	Immediately (injected)	Up to 8 weeks	↑ FS, ↑ Blood vessels	[61]
Algisyl	Clinical Study (NCT01311791)		(injected)	Up to 1 year	↑ Exercise capacity, = EF, = LVEDD, = LV function, size and mass	[64]
BCM	Clinical study (NCT01226563)		(injected)	Up to 6 months	= LV remodelling No clinical benefits	[65]
HA	Adult male Dorset sheep	LAD ligation	30 min (injected)	Up to 8 weeks	↑ LV wall thickness, = LVEDV, ↓ LVESV, ↓ LV dilation, ↑ EF	[69]
Starch	Male SD rats	LAD ligation	Acute MI model			[70]
			Immediately (implanted)	Up to 12 weeks	↓ LVEDD, ↓ LVESD, ↑ EF, ↑ FS, ↑ LV wall thickness, ↓ Fibrosis, ↓ Infarct size, ↓ myocyte hypertrophy, ↓ Inflammation	
			Subacute MI model:			
			1 week (implanted)	Up to 3 weeks	= LVEDD, = LVESD, ↑ EF, ↑ FS, ↑ LV wall thickness, ↓ Fibrosis, ↓ Infarct size, ↓ myocyte hypertrophy	
SF	Male SD rats	Permanent LAD ligation	7 days (injected)	Up to 12 weeks	↑ FS, ↑ EF, ↑ LV wall thickness, ↓ Fibrosis, ↓ LV enlargement	[72]
CS	Male SD rats	Permanent LAD ligation	1 h (injected)	Up to 16 weeks	↓ Infarct size, ↑ Angiogenesis, ↓ LVEDD, ↓ LVESD, ↑ EF	[77]
Acetylated CS	Male Wistar rats	Permanent LAD ligation	4 weeks (implanted)	Up to 4 weeks	↑ EF, ↑ FS, ↓ LVESV, ↓ LVEDV, = Capillaries number per cardiomyocytes, ↓ Fibrosis, = Proinflammatory cytokines expression	[78]
Poly(NIPAAm-co-HEMA-co-MAPLA)	Female Yorkshire swine	Permanent LAD ligation	3 weeks (injected)	Up to 8 weeks	↑ EF, = LVEDV, = LVESV, ↑ FAC, ↓ Scar size, ↑ LV wall thickness, ↑ LV stiffness, ↑ Cell infiltration, ↑ Tissue integration, ↑ Vascular maturation	[79]
Dex-PCL-HEMA/PNIPAAm	Male SD rats	Permanent LAD ligation	Immediately (injected)	12 weeks	↓ LVEDD, ↓ LVESD, ↓ LVEDP, ↑ FS, ↓ Cardiac hypertrophy ↑ Scar thickness, ↓ Infarct size, ↓ Fibrosis	[80]
Gelatin methacryloyl	C57BL6 mice	Permanent LAD ligation	Immediately (implanted)	3 weeks	↑ LV anterior wall thickness, ↓ LV posterior wall thickness, = LVEDD, = LVESD, ↑ FS, ↓ Scar formation	[81]
**Conductive scaffolds**						
rGO/silk	Male SD rats	Permanent LAD ligation	Immediately (implanted)	4 weeks	↑ EF, ↑ FS, ↑ Angiogenesis, ↑ Cardiomyocyte survival, ↑ Contractile function, Resistance to ventricular fibrillation	[87]
PAMB-G hydrogel	Female SD rats	Permanent LAD ligation	1 week (injected)	4 weeks	↑ FS, ↑ EF, ↓ LVEDD, ↓ LVESD, ↓ Scar Size, ↑ Scar thickness, ↓ Immune cells infiltration, ↑ Electrical conduction, ↓ Arrhythmias, Synchronises cardiomyocyte contraction	[88]
HA-CHO/HHA	Male SD rats	LAD ligation	Immediately (injected)	4 weeks	↑ FS, ↑ EF, ↓ LVEDD, ↓ LVESD, ↓ LVESV, ↓ LVEDV, ↑ Electrical conduction, ↓ Fibrosis, ↓ Infarct sizes, ↑ Wall thickness, ↑ Angiogenesis	[89]
GelDA/DA-Py			Immediately (painted)			
PPY-HI	Female SD rats	LAD ligation	1 week (injected)	Up to 12 weeks	↑ FS, ↑ EF, ↓ LVESV, = LVEDV, ↑ Angiogenesis, ↓ Fibrotic scar resistivity, ↓ QRS/QT intervals, ↓ Arrhythmia, ↑ Electrical conduction, Synchronise cardiomyocyte contraction.	[90]
(GO-Au)-CS	Male Wistar rats	LAD ligation	Immediately (implanted)	Up to 5 weeks	↑ Conductivity, ↑ Conduction velocity and contractility, ↑ QRS interval, ↑ EF, ↑ FS, ↑ LVEDD, = LVESD	[91]
GO-OPF	Male SD rats	LAD ligation	Immediately (injected)	Up to 4 weeks	↓ Infarct sizes, ↑ Wall thickness, ↑ FS, ↑ EF, ↓ LVESV, = LVEDV, ↑ Neovascularisation, ↑ Electrical conductance, ↑ Cardiac repair	[92]
Gelatin/Pyrrole-dopamine	Male SD rats	LAD ligation	Immediately (painted)	4 weeks	↑ FS, ↑ EF, ↓ LVESV, ↓ LVEDV, ↓ QRS interval, ↓ Fibrosis, ↓ Infarct sizes, ↑ Wall thickness, ↑ Revascularisation	[93]

(↑: Increase, ↓: Decrease, =: No significant variation, MI: Myocardial Infarction, SD: Sprague Dawley, LAD: Left Anterior Descending artery, ECM: Extracellular Matrix, LV: Left Ventricular, EF: Ejection Fraction, EDA: End Diastolic Area, ESA: End Systolic Area, FAC: Fractional Area Change, FS: Fractional Shortening, LVEDD: LV end-diastolic diameter, LVEDV: LV end-diastolic volume, LVESD: LV end-systolic diameter, LVESV: LV end-systolic volume, LVEDP: LV end-diastolic pressure VT: Ventricular Tachycardia).

## 3. Functionalised Acellular Scaffolds

Recently, many studies have focused on the implementation of acellular scaffolds via functionalisation with various compounds/strategies to improve their effectiveness adding new features, properties, abilities and functions in a controlled way (Table 2).

### 3.1. Acellular Scaffolds Coupled with ECM Components

In light of the importance of the ECM signals in driving the repair/regeneration of the infarcted heart, a few studies exploited conjugation between polymeric scaffolds and ECM components. In a study, the influence of integrated ECM along with mechanical anisotropy on scaffold function was investigated in a rat model of chronic MI employing three microfibrous poly (ester carbonate urethane) urea (PECUU) scaffold types that included longitudinal group, orthogonal group and ECM group [94]. Longitudinal group scaffolds were tailored with non-physiological mechanics and were fabricated to align their stiffer orientation longitudinally to cardiac tissues. Orthogonal group scaffolds imitated LV native mechanics and were designed to align their stiffer orientation parallel to the heart circumferential direction. In ECM group scaffolds polymer fibres were placed isotropically to provide isotropic mechanics and scaffolds were functionalised to contain a decellularised porcine cardiac ECM supplemented layer to face the epicardium. The results after ten weeks of scaffolds epicardial placement depicted numerous functional outcomes in ECM group scaffolds that included mitigation of maladaptive ventricular remodelling, progression in angiogenesis, attenuated LV wall thinning and scar formation, therapeutic mechanical support to left ventricle and hampered functional worsening. However, the impact of scaffold orientation (longitudinal and circumferential) was not promising; after eight weeks longitudinal and circumferential groups displayed comparable results probably because of scaffold fibrous encapsulation as a result of host foreign body response. Moreover, scaffolds containing ECM components were therapeutically more active compared to patches providing sole mechanical support and stiffness. In brief, the study has demonstrated that scaffolds providing mechanical support along with incorporated ECM components are more beneficial as compared to scaffolds providing mechanical support alone; indeed the only fibres mechanics changes, without incorporation of ECM components, had no significant functional outcomes [94]. This study has illustrated the significance of coupling ECM components with biodegradable polymers to synthesise a hybrid biomaterial. However, in this study, ECM incorporated scaffolds with altered mechanics were not studied. Such studies could highlight more clearly the functional impact of scaffold architectural orientation along with exogenous decellularised ECM on infarcted hearts.

Most of the studies in cardiac engineering address remodelling time points soon following MI. This acute strategy has very low significant relevance as much remodelling in the clinical scenario takes place unnoticed over a longer time frame that leads towards progressive cardiac failure [95]. A very recent study has aimed to address the critical question of whether after extensive remodelling and functional loss have taken place it is still relevant to apply a therapeutic patch [96]. This study has highlighted that biohybrid patches made of PECUU and decellularised ECM when applied over severely impaired chronic infarcts in rats after much adverse remodelling had taken place induced positive remodelling signatures. PECUU-ECM biohybrid scaffolds integrate the biodegradable polymer possessing mechanical strength and high anisotropy with decellularised ECM encompassing bioactive moieties such as matricellular proteins and growth factors. PECUU-ECM patch was implanted on LV infarct after eight weeks of chronic MI infarction with extensive adverse remodelling and then follow up was done at the end point of eight weeks (sixteen weeks post-MI). The results showed significant improvement in echocardiographic endpoints, improved EF and FS, smaller infarct size, stiffer left ventricle, and reduced LV dilation. In this study, the experimental design attempts to reproduce extensive adverse remodelling as it addresses ischemia eight weeks post-MI [96]. Therefore, this strategy may offer great potential to be clinically translatable in chronic infarction patients.

### 3.2. Acellular Scaffolds Coupled with Exogenous Cardioprotective Compounds

Many studies on MI pointed out the signalling pathways and related molecular factors involved in cardioprotection [97].

In a pre-clinical porcine model, a microstructured PLGA/gelatine 3D cardiac patch with cardioinductive properties functionalised with adenosine was tested in in vivo ischaemic heart. It was shown to suddenly induce activation of the pro-survival RISK pathway that limited the myocardial injury. In fact, after three months, higher preservation of the myofibrillar structure of CMs accompanied by decreasing inflammation and fibrosis was detected [98]. These functionalised scaffolds showed the potentiality to allow exogenous compounds to reach the injured myocardial tissue even in the absence or limited coronary flow and, at the same time, to restrain systemic adverse effects. However, since this feasibility study was limited to a few animals only, further detailed studies in large pre-clinical models are required to confirm and completely understand its therapeutic role, before employing it in a clinical scenario.

In a very recent investigation, another research group produced a scaffold coupled with nitric oxide (NO), another cardioprotective agent [99]. Exogenous NO delivery exhibits its cardioprotective effect against MI injury, acting on CMs survival and improving their contractility. NO is also a signalling molecule involved in inflammatory response modulation, platelets aggregation inhibition, and vascular tone relaxation [100,101]. Zhu et al., fabricated biodegradable PCL patches that were covalently linked with nitrate moieties, which enabled site-specific NO release in an infarcted microenvironment. Interestingly, implantation of these patches presented therapeutic benefits that included reduced injury, attenuated LV remodelling and restoration of cardiac function in both acute and chronic rat MI models. Further, the therapeutic potential of these patches was translated into a large animal model of acute MI where they demonstrated suppressed adverse LV remodelling. Interestingly, besides enhanced cardiac function and decreased infarct size, this study reported a significant increase in CMs proliferation at the border zone of the infarct size accompanied by enhanced capillary and arteriolar density [99].

It is noteworthy that the abundant production of reactive oxygen species (ROS) plays a critical role in LV remodelling after MI [102]. Ding et al., fabricated a hydrogel tailored by co-polymerisation of ROS-cleavable and consumable hyperbranched polymers (HBPAK) and methacrylate HA (HA-MA). This hydrogel entrapped with catalase can act as a ROS scavenger and O_2_ generator in the infarcted microenvironment to impede inflammation and relieve hypoxia. In the rat MI model, this hydrogel displayed a reduction in apoptosis and infarct area, inhibition of inflammation, stimulation of angiogenesis, and almost full recovery of cardiac function [103]. Other researchers coupled the scaffold with Bioglass, a cardioprotective compound that suppresses oxidative stress and possesses angiogenic properties, thus showing potential to promote vascularisation and tissue repair [104]. Bioglass loaded in sodium alginate hydrogel has shown to attenuate LV remodelling in rat MI model by inducing angiogenesis, reducing apoptosis and scar size as well as improving ventricular function [105].

### 3.3. Acellular Scaffolds Coupled with Growth Factors

Growth factors have gained attention in the treatment of cardiopathies due to their direct response to various cellular functions such as migration, proliferation and adhesion. It is noteworthy that tissue regeneration is an important healing step post-MI. In this context, activation of endogenous resident cells and mobilisation of progenitor cells to the infarcted site has gained strong recognition as it can trigger the neovascularisation process and endogenous regeneration. Indeed, one of the hostile hurdles in post-MI recovery is angiogenesis impedance in the infarcted area thus leading to hypoxia and cell death. In this paradigm, growth factors can contribute to vasculogenesis and cardiac repair to evade HF. Formerly, the delivery approach commonly used was to infuse growth factors into systemic circulation; however, a major concern it encountered was the limited myocardial uptake and short half-life [106]. For this reason, scaffold-based approaches that allow the local, controlled and continuous release of growth factors along with mechanical support to the myocardial tissues have gained much appreciation.

The insulin growth factor-1 (IGF-1) plays a relevant role in the recruitment of endogenous stem/progenitor cells towards injured regions and stimulates angiogenesis [107]. Substance P (SP), a neuropeptide, possesses the potential to modulate the inflammatory response and induce neovascularisation [108]. A notable pre-clinical study has employed polycaprolactone/collagen type 1-based patches coupled with IGF active region-derived IGF-1C peptide and SP. When implanted in infarcted rodents in situ IGF-1C and SP behaved synergistically to recruit endogenous mesenchymal stem cells and promote blood vessel regeneration besides other therapeutic benefits [109].

Granulocyte colony-stimulating factor (GCSF) holds the characteristic to recruit stem cells from bone marrow and induce their differentiation towards the endothelial cells or CMs in contact with infarcted myocardium. This unique ability of GCSF fuelled the scientific interest to exploit its beneficial aspects in the treatment of various cardiovascular diseases [110]. Keeping in view this role of GCSF, Spadaccio et al., demonstrated the therapeutic impact of poly-L-lactide (PLLA) scaffold functionalised with GCSF in infarcted rabbits. These authors showed that GCSF functionalised scaffolds offered marked cellular colonisation within micrometric fibres as compared to non-functionalised scaffolds. Moreover, neo-angiogenesis was driven by the topography of the biopolymer, and this biological effect was associated with the improvement of cardiac function. In addition, increased connective tissue accumulation, reorganised ECM architecture and scar remodelling were evidenced [111].

The basic fibroblast growth factor (bFGF) is a strong growth factor that favours stem cell survival and induces neovascularisation [112,113]. bFGF coupled with gelatine hydrogel [114] and Dex PCL HEMA/PNIPAAm hydrogel [115] in infarcted rodent models has shown to promote angiogenesis and reduce infarct size. In the canine chronic MI model, bFGF laden in gelatine hydrogel has been shown to enhance vascular density along with improvement in LV function [116].

In numerous studies, scaffolds coupled with vascular endothelial growth factor (VEGF) have been documented to prevent adverse LV remodelling and recover cardiac function in infarcted rodents. VEGF coupled with alginate-CS hydrogel [117] and Dex-PCL-HEMA/PNIPAAm hydrogel [118] have been shown to promote angiogenesis and reduce infarct size. HA-based ECM-mimetic hydrogel coupled with VEGF and artificial apoptotic cells (AACs) that were created by customised liposomes have been shown to prevent cardiac hypertrophy and inflammation along with other therapeutic outcomes [119]. VEGF along with BMP9 laden in alginate-based composite hydrogel also reduce fibrosis and promote angiogenesis in infarcted hearts [120]. In a more recent study, the authors combined the delivery of VEGF and IGF-1 by a hydrogel composed of ureido-pyrimidinone (UPy) moieties coupled to Polyethylene Glycol (PEG). Through novel and unique triple-marker MRI approaches, it was assessed in the rat MI model that this hydrogel system has the potential to induce cardiac regeneration by inducing angiogenesis, inhibiting fibrosis and encouraging revascularisation [121].

Myeloid-derived growth factor (MYDGF), a paracrine-acting protein has been shown to recover cardiac function in infarcted hearts in pre-clinical studies [122,123]. Yuan and colleagues developed poly (PEG-co-citrate) (PPC)-ethyl ester of a thiol acid (ET) hydrogel encapsulated with MYDGF to deliver citrate and MYDGF to infarcted rat hearts and observed improved heart repair [124].

Neuregulin is an epidermal growth factor that acts as a signalling protein which bind to ErbB4 receptor expressed by CMs that results in activation of downstream signalling pathways which promote cardioprotective effects [125]. Cohen and colleagues reported that in a large animal MI model, neuregulin encapsulated in HA-based hydrogel has been shown to reduce infarct size and enhance post-infarct ventricular contractility [126].

Interestingly, Kontonika et al. used alginate-based scaffolds to deliver growth hormones (GH) in a rat model in which ischemia-induced arrhythmogenesis was induced to study its effect on ventricular tachycardia and heart rate variability. During an observation period of 24 h, ventricular tachycardia and sympathetic activation were attenuated while infarct size was unaffected [127]. However, these outcomes need further detailed investigations to completely understand the root pathways and mechanisms involved in the antiarrhythmic properties of growth hormones. Recently, Feng et al. developed an alginate-based composite hydrogel scaffold coupled with IGF-1 adsorbed on silk fibroin (SF) microspheres to allow controlled and sustained release of IGF-1. This hydrogel when injected in the MI rat model has been shown to improve cardiac function [128].

Steele et al. fabricated a shear-thinning, self-healing, bioengineered hydrogel (SHIELD) composed of a copolymer of PEG vinyl sulfone (PEG-VS) and customised peptide P1 to couple a protein-engineered dimeric fragment of Hepatocyte growth factor (HGFdf). This hydrogel has demonstrated synergistic therapeutic outcomes of SHIELD and HGFdf on ischemic hearts in rat models to limit post-infarction adverse LV remodelling [129]. Recently these authors demonstrated the therapeutic impact of two protein-engineered cytokines, HGFdf and engineered stromal cell-derived factor 1α (SDF-1α) encapsulated in hydrogel composed of HA and PEG-poly lactic acid (PLA) nanoparticles (NPs) in small and large preclinical models [130]. The therapeutic outcomes in the rat MI model were comparable to their previous study [129]. These encouraging therapeutics outcomes inspired the authors to translate this treatment into large animal models. Nevertheless, when applied in the sheep MI model the beneficial effects were limited to a reduction in infarct size [130].

On the basis that heart-derived cardiac stromal cells (CSCs) hold regenerative therapeutic effects by secreting paracrine mediators and interacting with CMs [131,132]. Huang and colleagues designed a new strategy to vehicle CSCs in the cardiac site of injury [133]. These authors fabricated acellular artificial cardiac patches (artCP) composed of decellularised porcine myocardial ECM scaffold coupled with human CSC-secreted factors encapsulated in biodegradable poly (lactic-co-glycolic acid) (PLGA). Among various factors released by artCPs, hepatocyte growth factor (HGF), IGF and VEGF-induced cell recruitment into the patch. In the rodent acute MI model, implantation of this scaffold boosted cardiac recovery by inhibiting scarring, encouraging angiomyogenesis and improving ventricular function. Moreover, biosafety and therapeutic potential were also confirmed in the porcine MI model, although in a small sample size, and the outcomes were aligned with results obtained in rodents [133]. Remarkably, this study also confirmed that freshly prepared artCP and cryopreserved artCP exhibited the same therapeutic potential hence overcoming the limitation of cell-based patches that are needed to be freshly developed to maintain cell viability, and making this product less vulnerable to storage and shipping conditions.

### 3.4. Acellular Scaffolds Coupled with Extracellular Vesicles

Extracellular vesicles (EVs) are membrane vesicles enclosing cytosol in the lipid bilayer that have originated from different subcellular compartments. EVs are heterogeneous populations secreted by cells and include exosomes and microvesicles [134]. EVs have garnered considerable attention due to their important contributions in paracrine signalling with angiogenic, proliferative, and anti-apoptotic outcomes [135]. EVs derived from endothelial progenitor cells (EPCs) possess the potential to improve cardiac function. Interestingly, direct acellular use of these EVs evades the concerns of cell engraftment or viability [136]. For precise localised and sustained delivery of EPC-derived EVs, a research group developed a hydrogel delivery approach by encapsulating EVs in HA shear-thinning gel (STG). This EVs loaded hydrogel was injected at peri-infarct in rat MI model and it was observed that EVs delivery via hydrogel enhanced therapeutic efficacy of EV-mediated myocardial preservation which included increased EV uptake by endothelial cells, enhanced peri-infarct angiogenesis, vascular proliferation and myocardial haemodynamic. Inflammatory cell recruitment was increased by both STG treatment and STG-EVs treatment but there was no significant difference between the two groups indicating that EVs addition did not increase inflammatory cell recruitment, but their effects were limited to coronary circulation [137].

EVs secreted by MSCs possess cardioprotective effects thus displaying the potential to treat MI [138]. Further, EVs can also be produced and collected in bulk from MSCs [139]. MSCs-derived EVs encapsulated in (RADA)4-SDKP, which is based on SAP hydrogel, is another cell-free scaffold that could possess the potential to treat MI. The results showed that treatment of rat hearts after acute MI with EVs in both free form or SAP hydrogel loaded forms improves cardiac function that includes low fibrosis, high angiogenesis and low inflammatory responses. In fact, no further effectiveness was seen when EVs were applied in conjunction with cardioprotective (RADA)4-SDKP hydrogel. One reason could be that in this study diluted (0.25%) SAP hydrogel was employed that can be more vulnerable to in vivo degradation [140]. In fact, when MSC-derived EVs were encapsulated in methacryloyl (Gelma), which cross-linked in situ [141] or in alginate hydrogel [142] thus conferring sustain delivery and retention in the infarcted tissue, the application of these EVs laden scaffolds has been shown to promote angiogenesis, improved cardiac function, reduce apoptosis and infarct size [141,142].

It should be kept in mind that EVs are enriched with a unique representative cargo of their origin cells. Therefore, the selection of cell types to derive and extract EVs is essential to determine the therapeutic window of EVs. It is noteworthy that iPS-CM-derived EVs are rich in cardiac-specific miRNAs that have the potential to modulate CM-specific processes. Actually, collagen hydrogel patch encapsulated with iPS-CM-derived EVs enables the extended release of EVs thus allowing direct and continuous treatment when implanted in infarcted rat hearts. Besides the other effects, in this case, the authors observed also the antiarrhythmic effect and reduced pathological hypertrophy [143].

### 3.5. Acellular Scaffolds Coupled with Peptides/Proteins

Many proteins are actively involved in tissue regeneration and repair. In this context, selective and specific modification of proteins or peptides can be employed to optimise biomaterial properties and biological effects.

Histone deacetylases (HDACs) are an enzyme family that regulates gene transcription by deacetylating key lysine residues on histones and playing a crucial function in recruiting various epigenetic regulators of gene promoters/enhancers on DNA, hence modulating various aspects of metabolic anomalies including cardiovascular pathologies [144,145]. 7-amino-acid peptide (7A, MHSPGAD) is one of the splicing variants of HDAC7 mRNAs that is encoded by its short open reading frame located at 5′ terminal. Previously in both femoral artery injury and hindlimb ischemia models, it was noticed that phosphorylated 7A (7Ap) displayed various therapeutic benefits that included enhanced stem cell migration, proliferation, and differentiation, increased angiogenesis, mitigated vascular injury and boosted recovery of blood perfusion [146]. Owing to its therapeutic benefits, Zhang and co-workers speculated the beneficial therapeutic contribution of 7Ap in MI therapy. They hypothesised that 7Ap could be efficiently delivered by ECM-derived collagen I hydrogel to the infarcted region to prevent LV remodelling and restore cardiac function post-MI [147]. Collagen I is an ECM protein that along with water makes 3D polymeric assembly. Previously, collagen I-based hydrogel had been used as scaffolds for delivery of various therapeutics owing to its high permeability, biocompatibility and ability to prolong retention and gradual release of biomolecules [148,149]. These authors tested their hypothesis in the mice MI model by injecting 7Ap-loaded collagen I hydrogel intramyocardially to the LV wall infarcted zone. The results obtained after two weeks of follow up included increased neo-microvasculogenesis, reduced apoptosis and enhanced CMs proliferation. In addition, the recruitment and differentiation of antigen-1-positive (Sca-1+) stem cells were increased in the infarcted region. Moreover, reduced LV wall fibrosis, restricted infarct wall thinning and improved heart function. These effects eventually attenuated adverse LV remodelling. These encouraging findings emphasised that 7Ap-collagen hydrogel can be considered for MI therapy [147].

McLaughlin and co-workers tailored injectable hydrogel matrices composed of recombinant human collagen type I (rHCI) and type III (rHCIII) that were crosslinked by N-ethyl-N-(3-dimethylaminopropyl) carbodiimide (EDC) and N-hydroxysuccinimide (NHS) and were glycosylated by glycosaminoglycan chondroitin sulphate C (CS). Interestingly, in situ assembly and cross-linking of these hydrogels enabled the formation of a biomimetic 3D matrix that promoted the polarisation of endogenous macrophages towards a pro-wound healing M2 phenotype, restored mechanical properties, reduced scar size, prevented heart enlargement and encouraged CMs survival to avert LV remodelling within infarcted myocardium in pre-clinical models. In comparison to rHCIII matrices, rHCI matrices were therapeutically more significant to avert LV remodelling when applied in the late proliferative phase post-MI [150].

Another research group employed sericin, a silk-derived protein used as a wound-healing agent, for the first time. Genipin-crosslinked sericin hydrogel, injected in the heart infarcted zone of mouse MI model, decreased scar production, improved LV wall thickness, promoted vasculogenesis, impeded inflammatory responses and reduced apoptosis, thus inducing a significant improvement in cardiac function and averting LV remodelling [151].

Recently, Feng et al. hypothesised that angiogenic peptides derived from VEGF have the potential to mimic the bioactivity of VEGF and can activate its receptor to promote angiogenesis. Their study tailored a scaffold composed of collagen and decellularised ECM to encapsulate angiogenic peptides [152]. Besides the positive effects on cardiac remodelling, peptides that mimic the biological growth factor effects, show potential advantages typical of small molecules such as lack of immunogenicity, limitation of side-effects, economical production, while the shorter half-life of this small molecule can be balanced by the prolonged scaffold release.

### 3.6. Acellular Scaffolds Coupled with Enzymes Inhibitors

After MI, a cascade of structural and biological events begins within the infarcted region. Disequilibrium in the proteolytic pathways in the infarcted region, such as the imbalance between matrix metalloproteinases (MMPs) and endogenous tissue inhibitors of metalloproteinase (TIMPs), is one of the biological events that contribute towards adverse LV remodelling. MMPs influence the remodelling course by contributing to ECM turnover and inflammatory signalling to remove necrotic CMs [153,154]. At the beginning, upregulated pro-inflammatory cytokines contribute to MMP activation; however, this long-term activation increases TIMP levels, ultimately unbalancing the MMP/TIMP ratio, which in turn affects LV remodelling processes [155]. After MI, the changes in TIMP levels influences proliferation and potential differentiation of cardiac fibroblast to myoblasts thus contributing towards ECM stability and remodelling [155,156]. Clinical studies pointed out a significant increase in plasma levels of MMPs in the early MI phase, which was also linked to adverse LV remodelling [157,158]. Though a few clinical studies have already targeted MMPs in MI patients, therapeutic outcomes were not completely clear due to issues related to limited sample size, a modest degree of LV remodelling, difficult targeted delivery and problematic dose up to significant therapeutic levels [159]. Indeed, targeting the MMP system to circumvent adverse LV remodelling post-MI holds therapeutic relevance.

Within this therapeutic framework, targeted delivery strategies could be a logical and promising approach. In current bioengineering approaches, therapeutic scaffolds could hold promise to effectively target proteolytic pathways such as the MMP system. In this context, a pre-clinical research study was performed using an HA-based MMP-sensitive hydrogel (HAMMPS) with recombinant tissue inhibitor of MMPs encapsulated into it, to target the myocardial ECM proteolytic system in large animal models. In this study, full-length recombinant protein (rTIMP-3) was incorporated into the hydrogel that encompasses MMP-cleavable peptide cross-links to control the rTIMP-3 release from the hydrogel. After 28 days, in the swine MI model, the implant of scaffold incorporating rTIMP-3 (HAMMPS/rTIMP-3) showed promising outcomes that included a 50% decrease in LV dilation and extent of MI wall thinning, attenuation in indexes of HF such as left atrial size and LV filling pressures as well as a significant decrease in transcriptional profile for myofibroblasts together with profibrotic pathways. In brief, the results highlighted that localised therapeutic delivery of MMP-sensitive scaffolds that release rTIMP to target myocardial matrix can improve LV function, architecture and remodelling after MI [160]. The effectiveness of MMP inhibitor in the improvement of cardiac function and attenuation of LV remodelling was also confirmed by the study of Fan et al. [161] applying in a rat MI model a peptide-based MMP-2 inhibitor CTTHWGFTLC (CTT) encapsulated in PNIPAAm copolymer hydrogel.

In other studies, this approach was also combined with growth factors/chemokines release. Awada and collaborators designed a composite hydrogel composed of fibrin gel and heparin-based coacervates loaded with TIMP-3, FGF-2 and SDF-1α [162]. In vivo, this hydrogel allows controlled and sustained release of TIMP-3 followed by FGF-2 and SDF-1α. When tested in rat MI model, this composite hydrogel has shown attractive therapeutic results to attenuate LV remodelling, improve cardiac repair and recover cardiac function by significant reducing in ECM degradation, fibrosis, apoptosis, inflammation and LV dilation and promoting angiogenesis, CM survival, stem cells recruitment and cardiac function [162]. In another investigation, a dual function glutathione-modified collagen hydrogel loaded with recombinant glutathione-S-transferase (GST)-TIMP-bFGF was studied in infarcted rat hearts. In this scaffold, MMP-2/9-cleavable peptide TIMP was enclosed between GST and bFGF [163]. Typically, MMP-2/9 is overexpressed in the infarcted region, thus allowing on demand-release of TIMP and bFGF at infarcted sites. This hydrogel scaffold has been shown to improve cardiac function by promoting vascularisation and attenuating LV remodelling [163].

Further broad investigations in pre-clinical studies could elucidate in more detail the therapeutic influences of TIMP in the infarcted zone. To date this strategy has been applied at injectable scaffold only; further study in which this new approach if extended to patches could be a promising tool for cardiac remodelling treatment.

### 3.7. Acellular Scaffolds Coupled with Drugs

Commonly the administration of the drugs in cardiac patients is done through a simple systemic delivery approach, which in general results in low efficiency, drug toxicity along off-target side effects [164]. Scaffolds coupled with drugs can overcome these limits owed to lower-dose drug-loaded scaffold and local drugs delivery at the infarcted site.

Colchicine (Col) is a natural anti-inflammatory drug that is used limitedly owing to systemic toxicity [165]. A study developed PLGA-EG-PLGA polymer hydrogel coupled with Col to maintain, sustain and control the local release of Col and evaluated its therapeutic impact in an infarcted mouse MI model. The results showed a reduction in cytokine release in the infarcted group with consequent attenuated inflammation, fibrosis and apoptosis, accompanied by increased cardiac function. Moreover, no considerable side effects including systemic toxicity were observed [166].

It is noteworthy that some drugs exert cardioprotective effects, such as Salvianolic acid B (SaB) [167], however, its limitation in clinical use is due to its fast decomposition that does not fit with the long-term MI therapeutic course. Polydopamine (PDA) enhances cross-linking tendency of hydrogels and possesses an active catechol group that can adsorb drug molecules efficiently [168]. A study tailored to deliver elastin-mimic hydrogels (EMHs) coupled with SaB-laden PDA NPs in infarcted rat hearts. This EMH/PDA-SaB hydrogel displays high retention time and prolonged release of SaB in the infarcted cardiac microenvironment. In infarcted rodent hearts, this hydrogel has been shown to recover cardiac function and mitigate negative LV remodelling [169]. However, it is well-known that cardioprotective drugs exert their actions when administered immediately after MI, thereby further studies are required to confirm its efficacy in sub-acute and chronic MI models.

Another study documented a nanodrug-loaded ROS sensitive hydrogel system composed of hyperbranched poly(β-amino esters) (HB-PBAEs), polydopamine deposited tanshinone IIA NPs (TIIA@PDA) and thiolate modified HA (HA-SH). HB-PBAEs and TIIA@PDA crosslinks with HA-SH when simultaneously injected in the infarcted heart, thus resulting in in situ hydrogel formation. This hydrogel in infarcted rat MI has been shown to improve ventricular function, increase LV wall thickness as well as reduce infarct size and inflammation [170]. Recently, a study has designed a multimodal drug-releasing microporous annealed particle (drugMAP) hydrogel [171]. It consists of forskolin, a cAMP activator, and Repsox, an inhibitor of TGF-β, which were loaded on NPs, then encapsulated into MMP sensitive PEG-based μGel beads. When injected, this hydrogel assembles in an infarcted rat heart, forming a contiguous porous drugMAP scaffold in situ. Interestingly, this mechanism is governed by endogenous factor XIIIa, which activates peptide K and peptide Q in μGel to encourage the binding among μGel beads. After five weeks, observations pointed out the promising effects of this scaffold in terms of the heart repair process, which involved tissue mechanical support, promotion of neovascularisation and cell migration whereas suppression of immune responses and fibrosis. This drugMAP system has added a novel platform for μGel-based scaffolds in MI therapeutic approaches and can have wide application in disease treatment options [171].

Methylprednisolone (MP) is a glucocorticoid drug that exhibits the potential to promote vascularisation and reduce inflammation, myocardial injury and collagen content in MI experimental models [172]. In this regard, Yao and co-workers reported that ROS responsive degradable polyurethane fibrous patches encapsulated with methylprednisolone have the potential to restore structures and functions of infarcted rat myocardium [173].

### 3.8. Acellular Scaffolds Coupled with Gene Therapy

Gene therapy is an attractive therapeutic strategy to counteract HF. However, its efficiency, safety and targeted delivery are still a matter of concern [174]. Among possible viral vectors, adeno associated viruses (AAV) are well reputed for their low pathogenicity and recombinant AAV vehicles have shown promising characteristics that include persistent transgene expression, transfection of both quiescent and dividing cells and low immunogenicity as compared to other viral vectors [175]. However, the ability of AAVs to transfect cardiac cells remains to be fully demonstrated.

Gu and collaborators employed scaffolds made of elastomeric polyester urethane urea (PEUU) and polyester ether urethane urea (PEEUU)-based patches loaded with AAV encoding reporter gene green fluorescent protein (GFP) to assess the transduction efficiency in rodents three days post-MI. Follow-up at 12 weeks evidenced that GFP protein expression could be observed in α-SMA- and cardiac troponin T-positive cells, thus suggesting targeted delivery of AAVs. Comprehensively, the study emphasised viral transduction and prolonged release of genes that, along with elastomeric mechanical support, provided evidence for the scaffold coupled with AAVs as a suitable therapeutic strategy for improvement of cardiac function post-MI. Specific genes of interest might be incorporated in AAVs in future work and their release kinetics tailored by coupling AAVs with scaffolds to target infarcted regions [176]. However, in this in vivo study, the transduction efficiency was limited, and this was attributed to low AAV patch loading and/or limited permeability of the epicardial cells. Further target genes should be identified and deepened pre-clinical studies are needed before implementing this promising genetic engineering approach in a clinical scenario.

On the other hand, miRs are an important evolutionary element to post-transcriptionally regulate gene expression by silencing mRNA that inhibits their translation and leads them towards degradation [177]. In response to MI, a disruptive imbalance of miRs takes place in the infarcted myocardium [178]. In recent years, miRs-based therapeutic approaches have accumulated considerable importance due to the easy production and synthesis of miR mimics and antagomiRs, with the additional benefit that they silence gene translation in the cytoplasm, thus requiring only cellular entry rather than nuclear entry which is comparatively more challenging [179,180]. Scaffolds loaded with miRs might have the potential to ensure their localised delivery in infarcted areas after MI to perform their therapeutic role. Various studies have been conducted in recent years that employed hydrogel scaffold-based delivery approaches for miRs for MI treatment in preclinical studies.

As miR-1825 acts as a key regulator to increase expression of miR-199a, a strong miR that promotes cardiac regeneration in rodents, Pandey and collaborators designed gelatine and silicate-based hydrogel loaded with AAV-miR-1825 and tested its therapeutic efficacy in the mice MI model [181]. Direct injection of AAV-miR-1825 alone in the peri-infarcted region failed in cardiac regeneration, while its efficacy was achieved when it was loaded into hydrogel, thus underlining scaffold relevance. In fact, hydrogel enabled a sustained viral release and prevented the immediate wash out of viral particles by the beating heart, thus enhancing the therapeutic output. The results obtained by AAV- miR-1825-hydrogel application on infarcted rat heart indicated promising functional benefits that included cardiac regeneration as indicated by endogenous CMs proliferation at the peri-infarcted site, prevention of ventricular dilation and adverse remodelling with consequent improvement in heart function [181]. In the prenatal and postnatal heart, the miR-302/367 cluster is involved in the regulation of CMs proliferation by silencing various genes involved in Hippo signalling which includes large tumour suppressor 2 (LATS2), Mps one binder (MOB) and macrophage-stimulating 1 (Mst1). It results in translocation of yes-associated protein, the transcriptional effector of the Hippo pathway, to the nucleus to stimulate gene pathways that foster cellular proliferation [182]. HA hydrogel loaded with miR-302 in infarcted myocardium of mouse MI model has shown a significant increase in pH3 and Ki67-positive CMs, indicating CM proliferation and functional regeneration in the infarcted border zone. Moreover, despite the vascular density not improving, an improvement of cardiac function after MI was evidenced as demonstrated by the increase in ventricular function [183]. However, apoptosis and infarct size did not change significantly compared to the control group.

In cardiac tissues, miR-29B is linked to the regulation of fibrosis and ECM remodelling due to its considerable role in collagen production [184]. It is well-documented that miR-29 family members target as many as 16 genes associated with ECM key proteins that include many collagen isoforms, integrin β1, MMP-2, elastin, fibrillin 1 and laminin γ. miR-29 is downregulated in many cardiac diseases thus suggesting its contribution to fibrosis development due to its relieved repression of ECM gene translation [185,186]. A hydrogel composed of thiolated HA crosslinked with PEG-diacrylate to deliver miR-29B was injected in the infarct border zone of the MI mice model. Among echocardiographic measurements, EF, FS and fractional area change were improved whereas there was no significant difference in LVESV and LVEDV along with scar area and heart mass. The mature/immature collagen fibres ratio was increased, and elastin was decreased at the infarct border zone, yet neither of them changed within the infarcted area. Blood vessel density was increased in both the border zone and within the infarcted area. In brief, the hydrogel loaded with miR-29B produced a localised therapeutic response, maintained cardiac function and significantly changed ECM maturity and organisation [187].

Based on the critical role of angiogenesis in the post-MI heart, some authors applied gene therapy to stimulate it, exploiting the miR-17-92 cluster [188]. miR-92a is one of the components of this cluster that negatively regulates endothelial cells and angiogenesis and its expression in ischemic myocardium is increased after MI [188]. Systemic administration of antagomiR-92a in infarcted mice heart promotes angiogenesis, repair of infarct tissue, increases cellular protection and impedes inflammation. These attractive results have given impetus to further explore miR-92a in post-MI treatment by developing an effective approach to deliver antagomiR-92a for optimally antagonising miR-92a. Gelatine hydrogel microsphere (GHM) sheet is a biodegradable gel that degrades slowly to release impregnated biomolecules gradually over fourteen days [189]. AntagomiR-92a impregnated GHM sheet when implanted in the peri-infarct zone in rats immediately after acute MI has been shown to increase capillary density in the infarct border area, promote cardiac stem cell accumulation, reduce infarct size, inhibit LV dilation and improve LV function. Sheet alone or the sheet impregnated with antagomiR-control show non-significant therapeutic outcomes [190]. The importance of stimulating the formation of new vessels in the infarcted heart was confirmed by Li and co-workers [191] who targeted antiangiogenic genes by using miR-21-5p which is highly expressed in endothelial cells [192]. These authors developed an injectable aldehyde-capped PEG (PEGCHO) hydrogel matrix as a delivery system for miR-21-5p adsorbed on mesoporous silica nanoparticles (MSNs). This delivery system enabled control, on-demand and sustained microRNA-21 release stimulated by the local acidic microenvironment. In the porcine MI model, it has been demonstrated that released MSNs have significantly impeded inflammatory responses and further MSNs assisted miR-21-5p delivery to endothelial cells has encouraged local neovascularisation and promoted CMs survival [191]. These anti-inflammatory and pro-angiogenic responses have synergistically contributed to narrowed infarct size. In brief, the hydrogel contributed to averting negative LV remodelling by improving cardiac function [191]. Further studies are required to highlight the effectiveness of this new scaffold when applied at a later timing as in chronic animal models.

These studies based on miRNA-based scaffolds as a therapeutic strategy for MI have highlighted that targeting miRNA have a high potential to treat ischemia and modulate different aspects of LV remodelling. It appears to be relevant to design further studies with longer follow-up windows to ascertain the maintenance of the relevant gene expression control.

**Table 2:** Summary of functionalised acellular scaffolds to treat post-MI damage in preclinical models.

Scaffold Composition	Delivered Molecules	Model	Protocol Followed to Induce MI	Timing of Scaffold Application Post-MI	Follow Up	Biological Effects	Ref.
Acellular scaffolds coupled with ECM components							
PECUU-ECM	ECM components	Female Lewis rats	LAD ligation	2 weeks (implanted)	Up to 8 weeks	↓ ESA, ↓ EDA, ↑ FAC, ↓ Scar formation, ↑ LV wall thickness, ↑ Angiogenesis	[94]
PECUU-ECM	ECM components	Female Lewis rats	LAD ligation	8 weeks (implanted)	Up to 8 weeks	↓ LV dilation, ↓ Infarct size, ↑ LV stiffness, ↑ EF, ↑ FS, = ESA, = EDA, = LV thickness	[96]
**Acellular scaffolds coupled with exogenous compounds**							
PLGA/gelatine	Adenosine	Female landrace pigs	Ischemia reperfusion	5 min (implanted)	12 weeks	↑ pro-survival RISK signalling pathways, ↓ Inflammation, ↓ Fibrosis	[98]
PCL	Nitric Oxide	Male SD rats	Ischemia reperfusion	Acute MI model			[99]
				Immediately (implanted)	4 weeks	↑ EF, ↑ FS, ↓ LVEDD, ↓ LVESD, ↓ Apoptosis, ↓ Inflammation, ↑ Angiogenesis, ↑ CM survival, ↓ Collagen deposition	
				Chronic MI model			
				4 weeks (implanted)	4 weeks	↑ EF, ↑ FS, ↓ Infarct size, ↓ Cardiac dilation	
		Pigs	Ischemia reperfusion	Immediately (implanted)	Up to 4 weeks	↑ EF, ↑ FS, ↓ LVEDV, ↓ LVEDD, ↑ Cardiac Output, ↓ Infarct size, ↑ Neovascularisation, ↑ LV thickness, ↑ CM proliferation	
HBPAK-(HA-MA)	ROS scavenger and O_2_ generator	Male SD rats	LAD ligation	Immediately (injected)	4 weeks	↓ Apoptosis, ↑ Angiogenesis, ↓ Infarct size, ↓ Inflammation, ↑ EF, ↑ FS, ↓ LVEDV, ↓ LVESV	[103]
Sodium-Alginate	Bioglass	Male SD rats	LAD occlusion	7 days (injected)	4 weeks	↓ Apoptosis, ↑ Angiogenesis, ↓ Infarct size, ↑ EF, ↑ FS, ↓ LVEDD ↓ LVESD	[105]
**Acellular scaffolds coupled with growth factors**							
Polycaprolactone/collagen type 1-	SP and IGF-1C	Female BALB/c mice	Permanent LAD ligation	Immediately (implanted)	2 weeks	↑ EF, ↓ LVESV, ↓ LVEDV, ↓ Collagen deposition, ↓ Fibrosis, ↓ Apoptosis, ↑ Endogenous stem cells recruitment, ↑ LV wall thickness, ↑ Vascularisation	[109]
PLLA	GCSF	Male New Zealand white rabbits	Permanent LAD ligation	4 weeks (implanted)	2 weeks	↑ EF, ↑ FS, ↓ LVESV, = LVEDV, = LVEDD, ↓ LVESD, ↓ Infarct size, ↑ Angiogenesis, ↑ ECM reorganisation	[111]
Gelatin sheets	bFGF	Male rats (F344/NJcl-rnu/rnu)	Permanent LAD ligation	4 weeks (implanted)	4 weeks	↑ FS, ↑ FAC, = LVEDD, ↓ Scar formation, ↑ Angiogenesis	[114]
Dex-PCL-HEMA/PNIPAAm	bFGF	Male SD rats	LAD ligation	Immediately (injected)	30 days	↑ Angiogenesis, ↓ Collagen content, ↓ Infarct size, ↓ Apoptosis, ↑ EF, ↓ LVESD, ↓ LVEDD	[115]
Gelatin sheets	bFGF	Male beagles’ canines	LAD ligation	4 weeks (implanted)	Up to 4 weeks	= LVEDD, ↓ LVESD, ↑ FS, ↑ FAC, = EDA, = ESA, ↑ Vascular density	[116]
Calcium alginate-CS	VEGF	Female SD rats	Permanent LAD ligation	4 days (implanted)	Up to 4 weeks	↑ FS, ↑ Scar thickness, ↓ Scar area, ↑ Angiogenesis	[117]
Dex-PCL-HEMA/PNIPAAm	VEGF165	Male SD rats	LAD ligation	Immediately (injected)	30 days	↑ Angiogenesis, ↓ Collagen content, ↓ Infarct size, ↓ Apoptosis, ↑ EF, ↓ LVESD, ↓ LVEDD	[118]
Hydrazide-HA, ALD-HA and ALD-DS	AACs and VEGF	Male SD rats	LAD ligation	Immediately (injected)	4 weeks	↓ Cardiac hypertrophy, ↓ LVEDD, ↓ LVESD, ↑ LV wall thickness, ↓ Fibrosis, ↓ Inflammation, ↑ Revascularisation	[119]
Alginate/ SF7calcium gluconate	VEGF and BMP9	Male C57BL/6 mice	Permanent LAD ligation	Immediately (injected)	4 weeks	↑ Angiogenesis, ↓ Fibrosis, ↑ EF, ↑ FS	[120]
Ureido-pyrimidinone-PEG	VEGF and IGF1	Male OF1 mice	Reperfusion ischemic injury	Immediately (injected)	Up to 22 days	↑ Angiogenesis, ↓ Fibrosis, ↑ Remuscularisation, ↑ EF, ↓ LVESV, = LVEDV	[121]
PPC-ET/PEG	Citrate and Mydgf	Male SD rats	Permanent LAD ligation	Immediately (injected)	4 weeks	↑ EF, ↑ FS, ↓ LVESD, ↓ LVEDD, ↓ Infarct size, ↓ Fibrosis, ↑ LV thickness, ↑ Neovascularisation	[124]
HEMA-HA	Neuregulin	Male Dorset sheep	Permanent LAD ligation	10 min (injected)	8 weeks	= Heart rate, = Mean Arterial Pressure, ↑ EF, = LVEDV, = LVESV, ↑ Stroke work index, ↑ Cardiac power index, ↑ LV contractility, ↓ Infarct size	[126]
Alginate-hydrogel	GH	Wistar rats	Ischemia reperfusion	10 min (injected)	24 h	= Infarct size, ↓ VT-episodes, ↓ Sympathetic activation	[127]
SF-Alginate	IGF-1	Female SD rats	Permanent LAD ligation	10 min (injected)	Up to 4 weeks	↑ EF, ↓ Infarct size, ↑ LV wall thickness, ↓ Fibrosis	[128]
PEG-VS/P1	HGFdf	Adult male Wistar rats	Permanent LAD ligation	Immediately (injected)	4 weeks	↑ EF, ↑ FAC, ↑ Stroke volume, ↓ LVESV, ↓ LVEDV, ↓ Infarct size, ↑ Arteriole density	[129]
HA-PEG-PLA	HGFdf and SDF-1α	Adult male Wistar rats	Permanent LAD ligation	Immediately (injected)	4 weeks	↑ Vessel density, ↓ Infarct size, ↓ LVESD, ↓ LV systolic area, ↓ LV diastolic area, ↑ EF, ↑ FS, ↑ FAC	[130]
		Male Dorset sheep	LAD occlusion	Immediately (injected)	8 weeks	↓ Infarct size, = EF, = LVEDV, = LVESV, ↓ End diastolic mass ↑ End systolic mass	
Decellularised myocardial ECM	ECM components and synthetic cardiac stromal cells, HGF, IGF, VEGF	Female SD rats	Permanent LAD ligation	Immediately (implanted)	3 weeks	↑ EF, ↑ FS, ↓ Infarct size, ↑ Viable cardiac tissue, ↑ Wall thickness, ↑ Angiomyogenesis, ↓ Apoptosis	[133]
		Female Yorkshire pigs	Permanent LAD ligation	10 min (implanted)	1 week	↑ EF, ↑ FS, ↓ Infarct size, ↓ Fibrosis	
**Acellular scaffolds coupled with Extracellular vesicles**							
STG	EPCs derived EVs	Male Wistar rats	Ischemia reperfusion	Immediately (injected)	Up to 4 weeks	Maximum rate of systolic and diastolic pressure change, ↑ EF, ↓ LVESV, ↓ LVEDV, ↑ Vascular density, ↑ Inflammatory cell recruitment, ↓ Scar thickness	[137]
(RADA)4-SDKP	MSCs derived EVs	Adult male Wistar rats	Permanent LAD ligation	Immediately (injected)	Up to 4 weeks	↑ EF, ↑ FS, = LVEDD, ↑ Fibrosis, ↑ Angiogenesis, ↓ Macrophage infiltration	[140]
GelMA	MSCs derived EVs	Male C57 BL mice	LAD ligation	Immediately (sprayed)	4 weeks	↓ Apoptosis, ↑ Angiogenesis, ↓ Scar size, ↑ Infarct thickness, ↑ EF	[141]
Sodium alginate	MSCs derived EVs	Male SD rats	Permanent LAD ligation	30 min (injected)	4 weeks	↓ Inflammation, ↓ Apoptosis, ↓ Infarct size, ↑ Angiogenesis, ↑ Scar thickness, ↑ EF, ↑ FS, = LVEDD, ↓ LVESD	[142]
Collagen	iPS-CM derived EVs	Athymic nude SD rats	LAD ligation	Immediately (implanted)	Up to 4 weeks	↓ Arrhythmic burden, ↓ Cell hypertrophy, ↑ EF, ↓ LVEDD, ↓ LVESD, ↓ Apoptosis, ↓ Infarct size	[143]
**Acellular scaffolds coupled with peptides/proteins**							
ECM-derived collagen I	7Ap	Female C57/B6 mice	Permanent LAD ligation	Immediately (injected)	Up to 2 weeks	↑ Neo-vasculogenesis, ↓ Apoptosis, ↑ CM cycle progression, ↑ stem cells recruitment, ↓ LV wall fibrosis, ↓ wall thinness, ↑ LV Stroke volume, ↑ FS, ↑ EF, ↑ Cardiac output, ↓ LVEDD, = LVESD	[147]
EDC/NHS/CS	rHCI rHCIII	Female C57BL/6 mice	LAD ligation	7 days (injected)	4 weeks	↑ EF, ↑ FAC, ↓ LVESV, = LVEDV, ↑ Stroke volume, ↑ Cardiac output, ↓ Scar size, = Vascular density, ↑ Capillary density in the border zone, ↓ CM’s survival	[150]
Sericin-genipin	Sericin	Male C57BL/6 mice	Permanent LAD ligation	Immediately (injected)	Up to 6 weeks	↑ LV wall thickness, ↓ Scar thickness, ↑ FS, ↑ EF, ↓ LVEDD, ↓ LVESD, ↓ LVESV, ↓ LVEDV, ↓ Inflammatory response, ↑ Neovascularisation, ↓ Apoptosis,	[151]
Decellularised ECM/ Collagen	Angiogenic peptide derived from VEGF	Male SD rats	LAD ligation	Immediately (injected)	Up to 12 weeks	↑ EF, ↑ FS, = LVEDD, = LVESD, = LV anterior wall, = LV posterior wall, = Interventricular septum, ↑ Vascularisation, ↓ Apoptosis	[152]
**Acellular scaffolds coupled with enzymes inhibitors**							
HA	rTIMPs	Male pigs	Permanent LAD ligation	Immediately (injected)	Up to 4 weeks	↓ EF, ↑ LVEDV, ↓ LV dilation, ↓ LV thinness, ↓ Left atrial size, ↓ pulmonary capillary wedge pressure, ↑ Contractile function ↓ Transcriptional profile of myofibroblasts and profibrotic pathways	[160]
PNIPAAm copolymer	MMP-2 inhibitor peptide	Male SD rats	Lad Ligation	30 min (injected)	4 weeks	↓ LV dilation, ↑ Wall thickness, ↑ Collagen type III/I ratio, ↑ Myofibroblast density, ↓ Fibrosis ↑ FS, ↑ EF, ↓ LVESV, ↓ LVEDV	[161]
Fibrin/Heparin	TIMP-3, FGF-2, SDF-1α	Male SD rats	Permanent LAD ligation	5 min (injected)	Up to 8 weeks	↑ FAC, ↑ EF, ↓ LVESV, ↓ LVEDV ↓ LV wall thinness, ↓ Ventricular dilation, ↑ cardiac function, ↓ Fibrosis, ↓ Apoptosis, ↑ CM survival, ↑ Stem cell recruitment, ↓ Inflammation, ↑ Angiogenesis	[162]
Glutathione modified collagen	GST-TIMP-bFGF	Male SD rats	LAD ligation	Immediately (injected)	4 weeks	↑ FS, ↑ EF, ↓ LVEDD, ↓ LVESD, ↑ Wall thickness, ↓ Collagen deposition, ↑ Vascularisation	[163]
**Acellular scaffolds coupled with drugs**							
PLGA-PEG-PLGA	Col	Male C57BL/6 mice	LAD ligation	Immediately (injected)	4 weeks	↓ Inflammation, ↓ Fibrosis, ↓ Apoptosis, ↑ FS, ↑ EF, ↓ LVEDD, ↓ LVESD, ↓ Ventricular wall stiffening	[166]
EMH/ PDA NPs	SaB	SD rats	Coronary artery ligation	Immediately (injected)	4 weeks	↑ FS, ↑ EF, ↓ LVEDD, ↓ LVESD, ↓ Apoptosis, ↓ Fibrosis, ↑ LV wall thickness, ↑ Vascularisation	[169]
HB-PBAEs/HA-SH	Tanshinone IIA	Rats	Permanent LAD ligation	Immediately (injected)	Up to 4 weeks	↓ Ventricular dilation, ↑ FS, ↑ EF, ↑ LV wall thickness, ↓ LVESD, = LVESV, ↑ Heart rate, ↑ LVSP, ↓ Myocardial relaxation time, ↓ LV maximum upstroke velocity, ↑ LV maximum descent velocity, ↓ Infarct size, ↓ Inflammation	[170]
DrugMAP-PEG	Forskolin and Repsox	Female SD rats	Ischemia reperfusion	2 days (injected)	5 weeks	↓ Infarct size, ↑ Wall thickness, ↓ LVESV, ↓ LVEDV, ↑ EF, ↑ Cell infiltration, ↑ Angiogenesis, ↓ Fibrosis, ↑ Cell migration, ↑ Inflammatory response,	[171]
Poly(thioketal) urethane	Methylprednisolone	Male SD rats	LAD ligation	30 min (implanted)	Up to 4 weeks	↑ EF, ↑ FS, ↓ LVEDV, ↓ LVESV, ↓ LVESD, ↓ LV dilation, ↑ LV thickness, ↓ Infarct size, ↑ Revascularisation	[173]
**Acellular scaffolds coupled with gene therapy**							
PEUU-PEEUU	Recombinant AAV	Female Lewis rats	LAD ligation	3 days (injected)	Up to 12 weeks	↑ LV wall thickness, ↑ Cell infiltration, ↓ EDA, ↑ FAC, ↑ EF	[176]
Gelatine and silicate	AAV-miR-1825	Female C57BL/6 mice	Permanent LAD ligation	Immediately (injected)	Up to 4 weeks	↑ EF, ↑ FS, ↓ LV dilation, ↓ Infarct size, ↑ CM proliferation	[181]
HA	miR-302	Male C57BL/6 mice	Ischemia reperfusion	Immediately (injected)	Up to 4 weeks	↑ CM proliferation and regeneration at the infarct border zone, ↓ LVESV, ↓ LVEDV, ↑ EF, ↑ FS, = Infarct size, = Apoptosis, = Vascular density, ↓ LVEDD, ↓ LVESD	[183]
Thiolated HA-PEG diacrylate	miR-29B	C57BL/6 mice	Ischemia reperfusion	45 min (injected)	Up to 5 weeks	↑ EF, ↑ FS, = Scar area, = Heart mass, = LVESV, = LVEDV, = Collagen fibres and elastin between infarct, ↑ Collagen fibres at infarct border, = Collagen fibre orientation & quantity, ↓ Elastin at infarct border zone, = ECM proteins expression, ↑ Blood vessel density	[187]
Gelatine	Antagomir-92a	Male SD rats	Ischemia reperfusion	Immediately (implanted)	Up to 2 weeks	↑ Angiogenesis, ↑ Stem cells accumulation, ↑ Cardiomyogenesis, ↓ LVEDD, ↑ FS	[190]
PEG_CHO_/MSN	miRNA-21-5p	Male Yucatan mini pigs	LAD ligation	Immediately (injected)	Up to 4 weeks	↑ EF, = LVESV, = LVEDV, = LV posterior wall thickness, ↓ Fibrosis, ↓ Infarct size, ↑ Vascularisation, ↓ Inflammation	[191]

(↑: Increase, ↓: Decrease, =: No significant variation, MI: Myocardial Infarction, SD: Sprague Dawley, LAD: Left Anterior Descending artery, ECM: Extracellular Matrix, LV: Left Ventricular, EDA: End Diastolic Area, ESA: End Systolic Area, EF: Ejection Fraction, FAC: Fractional Area Change, FS: Fractional Shortening, LVEDD: LV end-diastolic diameter, LVEDV: LV end-diastolic volume, LVESD: LV end-systolic diameter, LVESV: LV end-systolic volume, VT: Ventricular Tachycardia), LVSP: ↑ LV systolic Pressure.

## 4. Future Directions and Conclusions

Acellular scaffolds are an attractive approach that has gained extensive attention in regenerative cardiac medicine, with a specific interest in the limitation of adverse LV remodelling. The major advantages of acellular scaffolds are immediate implantability and customisable morphological design for stimulation of tissue regeneration, providing integration and tuned mechanical support at infarcted sites. In addition, acellular scaffolds can be easily produced, transported and stored, and for these reasons, there is a strong interest from the biomedical companies for this solution.

Ideally, an acellular cardiac patch has to contain the necessary regulatory signals to stimulate precise reactions at a molecular level to direct cell behaviour in terms of stem cell recruitment, cardiac and/or endothelial differentiation and electrical coupling between adjacent cells to recapitulate developmental processes in morphogenesis. A relevant future direction is to further impinge on the ability of the engineered scaffold to crosstalk with resident cells in a precise and controlled manner through spatial and temporal control as this is a critical requirement. Biodegradable polymeric materials are particularly suitable to be processed using different microfabrication techniques to obtain micro-nano topography mimicking the anisotropic structure of the myocardium providing a spatial interface to control adhesion, elongation, ordered disposition and myocardial commitment of stem cells.

**Figure 2:**
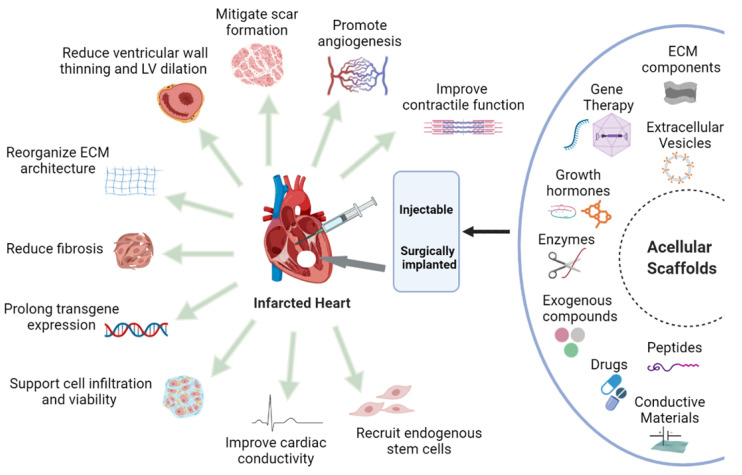
Acellular scaffold strategies applied in the infarcted heart to overcome myocardial infarction-induced LV remodelling. On the (**right**), properties and functionalisations of several acellular scaffolds, that can be either injected or implanted on an infarcted heart, are illustrated. On the (**left**), the biological effects obtained by various types of scaffold functionalisation on LV remodelling are shown. (ECM: Extracellular Matrix; LV: Left Ventricular).

Other emerging directions are the concept of open scaffold platforms that can be adapted to the patients’ needs and a better search for mininvasivity. Acellular natural and synthetic polymeric scaffolds can be combined with drugs, specific molecular signals or nanosystems to achieve cardiac tissue repair and sustain cardiac function in the long term (Figure 2). Recently, also 3D natural or synthetic hydrogels can be fabricated to resemble native tissue and incorporate signals molecules and drug delivery systems, however, their biomechanical integrity often does not achieve stiffness of the myocardium. Furthermore, the structural reproducibility of hydrogels is not yet guaranteed, thus reducing the possibility of maintaining spatial geometries and specific orientation of biological signals. Several injectable biomaterials have already been employed in pre-clinical investigations in both MI and HF models, however, only a few of them can be delivered via a catheter due to unique material design constraints required. The control of viscosity during insertion and the application of physical or chemical crosslinking methods in situ can be difficult and this, together with lack of hemocompatibility unfortunately prevents most injectable biomaterials from this type of minimally invasive delivery procedure, that would indeed represent an important clinical advantage.

A well-reasoned choice of relevant pre-clinical and clinical models is another future challenge. The research in this field is still far limited to pre-clinical models and most of the studies consisted of implant or injection of scaffolds shortly after MI induction, conditions difficult to reproduce in clinical scenarios. A deeper knowledge about the timing of intervention is needed, as for the medical treatments also the right time of surgery plays a key role in the improvement of post-infarct patient outcomes. For this reason, more studies to be performed in vivo chronic models are required. The experiments should be performed also when the cardiac remodelling has already developed, thus mimicking the timing of intervention in the post-MI patients by the clinicians/surgeons. Further, extensive research is still required for monitoring the fate of implanted scaffolds in pre-clinical studies to evaluate their long-term impact on cardiac function. For this reason, a joint effort should be made by both researchers and clinicians to achieve suitable and well-designed experimental protocols that could meet surgical requirements, to obtain fundamental knowledge that may allow one to transfer this biomaterial-based strategy from bench to bedside.

After several years of investigation in this field, it can be concluded that besides mimicking myocardial properties, the acellular scaffold should be functionalised with several strategies to achieve the multiple desired benefits, including a reduction of scar extension, formation of new vessels, as well as an improvement in cardiac function and electrical conductivity. The results of the reported studies highlighted that the specific properties of the scaffolds can impact different aspects of cardiac remodelling, thus suggesting that only combinatorial approaches will allow synergistic beneficial effects. The current knowledge and progress on acellular scaffolds will pave the way to plan effective approaches for the construction of clinically operative acellular scaffolds and direct the research towards humans to assess their clinical success.

Remarkable preclinical advances have been made for the use of acellular scaffolds alone or in combination with biotherapeutic moieties to limit LV remodelling post-MI. This compelling progress in the field of biomaterials and cardiac engineering of therapeutic acellular scaffolds can be considered a very attractive option for MI treatment in the clinical setup in future. Further in-depth investigation on the mechanism of action of acellular scaffolds will provide further novel cues for the customised engineering of these acellular scaffolds. Although it is just the beginning of this therapeutic approach, the large quantum of efforts made till now has provided noteworthy clues to rapidly accelerate further developments.
